# Joint analysis of scATAC-seq datasets using epiConv

**DOI:** 10.1186/s12859-022-04858-w

**Published:** 2022-07-29

**Authors:** Li Lin, Liye Zhang

**Affiliations:** School of Life Science and Technology, ShanghaiTech University, Shanghai, China

**Keywords:** scATAC-seq, Cell clustering, Batch effects, Data integration

## Abstract

**Background:**

Technical improvement in ATAC-seq makes it possible for high throughput profiling the chromatin states of single cells. However, data from multiple sources frequently show strong technical variations, which is referred to as batch effects. In order to perform joint analysis across multiple datasets, specialized method is required to remove technical variations between datasets while keep biological information.

**Results:**

Here we present an algorithm named epiConv to perform joint analyses on scATAC-seq datasets. We first show that epiConv better corrects batch effects and is less prone to over-fitting problem than existing methods on a collection of PBMC datasets. In a collection of mouse brain data, we show that epiConv is capable of aligning low-depth scATAC-Seq from co-assay data (simultaneous profiling of transcriptome and chromatin) onto high-quality ATAC-seq reference and increasing the resolution of chromatin profiles of co-assay data. Finally, we show that epiConv can be used to integrate cells from different biological conditions (T cells in normal vs. germ-free mouse; normal vs. malignant hematopoiesis), which reveals hidden cell populations that would otherwise be undetectable.

**Conclusions:**

In this study, we introduce epiConv to integrate multiple scATAC-seq datasets and perform joint analysis on them. Through several case studies, we show that epiConv removes the batch effects and retains the biological signal. Moreover, joint analysis across multiple datasets improves the performance of clustering and differentially accessible peak calling, especially when the biological signal is weak in single dataset.

**Supplementary Information:**

The online version contains supplementary material available at 10.1186/s12859-022-04858-w.

## Background

The expression of genes is regulated by transcription factors (TFs) that bind to the regulatory elements of the genome. As the accessible chromatin covers more than 90% TF binding regions, many techniques, such as Assay for Transposase-Accessible Chromatin using sequencing (ATAC-seq), have been developed to detect accessible chromatin [1–3]. Technical advancements have made it possible to profile the chromatin states of single cells at a high-throughput manner and along with other molecular modalities (e.g. transcriptomes) in the same cells [4–6]. In recent years, several studies have put efforts to profile chromatin landscapes of organisms at single-cell resolution and generated atlas of datasets across diverse species, tissues and conditions [7, 8]. Joint analysis on these data will help us better learn the roles of chromatin states under healthy and disease conditions.

Similar to RNA-seq, ATAC-seq data from multiple sources routinely shows strong technical variations and suffers from batch effects [9], which limits large-scale analysis across datasets. Batch correction on scATAC-seq data is more challenging as it is difficult to capture and correct batch derived variations on nearly binary chromatin profiles. Most clustering algorithms for scATAC-seq data capture biological information by learning a series of latent features from binary chromatin profiles. As most benchmarking studies up to now are performed on datasets with little batch effects [10, 11], whether these clustering algorithms are confounded by batch effects is unknown. In scRNA-seq analysis, batch effects can be removed by linear correction on a set of gene expression-derived meta features (e.g. Principal Components) [12–14]. However, it remains unclear whether batch effects of scATAC-seq can be corrected similarly on latent features by existing tools.

Here, we develop a novel algorithm named epiConv (https://github.com/LiLin-biosoft/epiConv) to remove batch effects between scATAC-seq datasets with better performance than existing tools. We find that latent feature learning algorithms benchmarked in existing studies (cisTopic [11], Latent Sematic Indexing (LSI) [4], SCALE [15] and SnapATAC [16]) all suffered from batch effects and in some cases, they could not be removed by linear correction using existing tools for scRNA-seq (Harmony [12], Mutual Nearest Neighbors (MNN) [14] and Seurat [17]). Unlike existing methods, epiConv directly calculates the similarities between cells without embedding them into the latent feature space. Although this strategy performs similarly compared to other methods in single cell clustering, subsequent batch correction on the similarity matrix better removes batch effects and, in the meantime, retains biological signals. Besides batch correction, we also demonstrate that epiConv can be used to analyze co-assay data through data integration. Aligning co-assay data to high quality scATAC-seq reference significantly increased the resolution of chromatin profiles and revealed thousands of putative connections between cis-regulatory elements and their target genes. Finally, we showed that integration of single cells in different biological conditions (T cells from germ-free and normal mouse, normal vs. malignant hematopoiesis) reveals the hidden heterogeneities between cells that cannot be detected from single dataset.

## Results

### Batch correction algorithms of epiConv

**Fig. 1:**
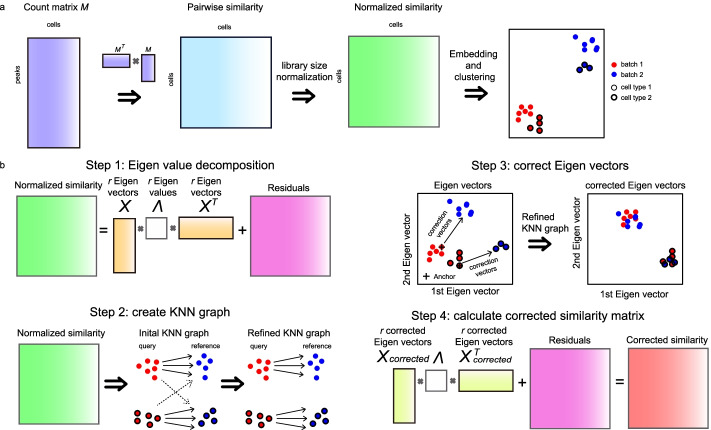
An overview of epiConv algorithm. **a** Workflow of calculating similarity matrix between single cells. The similarity matrix is calculated by matrix product of feature by cell matrix *M* and is normalized by library size. Downstream analyses are applied on normalized similarity matrix. **b** Workflow of batch correction. EpiConv removes the batch effects through linear correction on the *r* Eigen vectors of the similarity matrix.

EpiConv first calculates the similarity matrix *S* between single cells from a binarized matrix *M*, where rows represent peaks and columns represent cells. The similarity between two cells is calculated by the dot product of two cell vectors, which means that the similarity matrix is calculated by matrix product ${M}^{T}M$. After normalization by library size, the similarity matrix can be used for other downstream analyses (Fig. 1a). A detailed description of this step can be found in Supplementary Note. In datasets with no batch effects, epiConv performs similarly or slightly better compared to existing algorithms (Additional file 1: Fig. S1). The result suggested that the relationships between single cells can be directly learned from the binary accessibility profiles using simple approach.

Up to now, most batch correction tools can only be applied to cells embedded in Euclidean space, which is not available in our algorithm. To overcome this problem, we develop an approach to remove batch effects on pairwise cell-cell similarity matrix (Fig. 1b). In the first step of batch correction, we apply Eigenvalue Decomposition to the normalized similarity matrix *S* and keep *r* Eigen values (diagonal elements of *Λ*) with the largest absolute values and their corresponding Eigen vectors *X* (Fig. 1b, Step 1). *X* capture the major variations of the data (including biological and technical variations) and their contributions to the similarity matrix are weighted by *Λ*. The number of Eigen vectors *r* depends on the complexity of data and an appropriate *r* can be easily obtained by manually examining the batch effects on Eigen vectors. In this study, we set *r *= 30 unless otherwise mentioned.

If the dataset is large (e.g. ~ 100k cells), we first sample *m*_1_ cells and calculate the Eigen vectors of sampled cells *X*_*sample*_ and *Λ*. The Eigen vectors of *m*_2_ unsampled cells *X*_*unsample*_ is calculated by the least square solution of the equation $S^{\prime } = X_{sample} \Lambda X_{unsample}^{T}$, where *S’* ϵ *m*_1_* x m*_2_ is the similarity matrix between *m*_1_ sampled and *m*_2_ unsampled cells. Such approximation has little effects on the Eigenvalue Decomposition if *m*_1_ is large enough to cover all cell types but reduces the memory requirement and running time for large datasets.

Second, we create a k-nearest-neighbor (KNN) graph between datasets (Fig. 1b, Step 2). For simplicity, assume that we have one query dataset *A* and one reference dataset *B*. For one cell *a* in *A*, we find its *k*_1_ nearest neighbors in *B* (*b*_1_*, b*_2_*,…*,*b*_*k*1_). The initial *k*_1_ nearest neighbors may contain cells that don’t share the same cell type as *a* (dash lines in Fig. 1b Step 2) when the corresponding cell type is not available in *B* or simply due to noise. We introduce a filtering step to remove them. For each cell *b* in *B*, we find its *k*_2_ nearest neighbors in *A* and calculated the mean and standard deviation (*u*_*b*_ and *σ*_*b*_) of them for each Eigen vector and Principal Component of RNA-seq (if *A* is multi-omics data). If *a* in *A* finds a neighbor *b*_1_ in *B* but *a* is out of the range *u*_*b*1 _± 2*σ*_*b*1_ in any Eigen vector or PC of RNA-seq (In dataset *A*, there are some cells more similar to *b*_1_ but differing from *a*), we remove *b*_1_ from *a*’s neighbors.

After filtering, some cells in *A* may still keep several neighbors in *B* while others may lose most of its initial neighbors. Cells in *A* that have enough neighbors in *B* (≥ 5 in this study) are used as anchors to calculate correction vectors (Fig. 1b). For each anchor in *A*, we calculate the correction vectors between itself and its neighbors in *B* (Fig. 1b, Step 3). The correction vectors of other non-anchor cells are calculated by the weighted mean of its most similar anchors (10 anchors in this study) on a shared-nearest-neighbor graph (SNN graph) of *A*, where the weight is equal to the edge of SNN graph. The SNN graph can be calculated from ATAC-seq or combined with RNA-seq profiles (if available).

Finally, the Eigen vectors of all cells in *A* are corrected accordingly and the similarity matrix is re-calculated from *X*_*corrected*_ (Fig. 1b, Step 4). Notably, retaining the residuals of Eigenvalue decomposition is an important step in batch correction. Although residuals in dimension reduction procedure are generally considered as noise and are removed in most methods, we find that directly inferring the relationships between cells in the reduced dimension of Eigenvectors like existing methods results in partial correction and lower accuracy (see Additional file 1: Fig. S9c, d in hematopoiesis dataset below), implying that the residuals contain biological signals.

EpiConv can also perform integration of multiple reference datasets and multiple query datasets. When multiple references and query datasets are available, we first integrate all references together (project Ref *B* to Ref *A*, then project Ref *C* to combined dataset containing A and B, …) and then align each query dataset to the combined dataset containing all references.

We used Uniform Manifold Approximation and Projection [18] (UMAP) in R package umap to learn 2D embeddings of single cells and Louvain algorithm implemented in R package Seurat [17] to cluster cells. In order to reduce noise, the similarity matrix between cells is first transformed to SNN graph (*S*_*snn*_) described above, with number of nearest neighbors set to 1% of total cells. Then, the distance matrix (Max (*S*_*snn*_) − *S*_*snn*_) is used as UMAP input. The Louvain clustering is performed on the transformed UMAP graph *G* from the distance matrix (Max (*S*_*snn*_)  − *S*_*snn*_) to generate consistent results between dimension reduction and clustering. All unmentioned settings are set to default values and the resolution setting in Louvain clustering is manually adjusted between 0.2 and 2.0.

### EpiConv is less prone to over-fitting

**Fig. 2:**
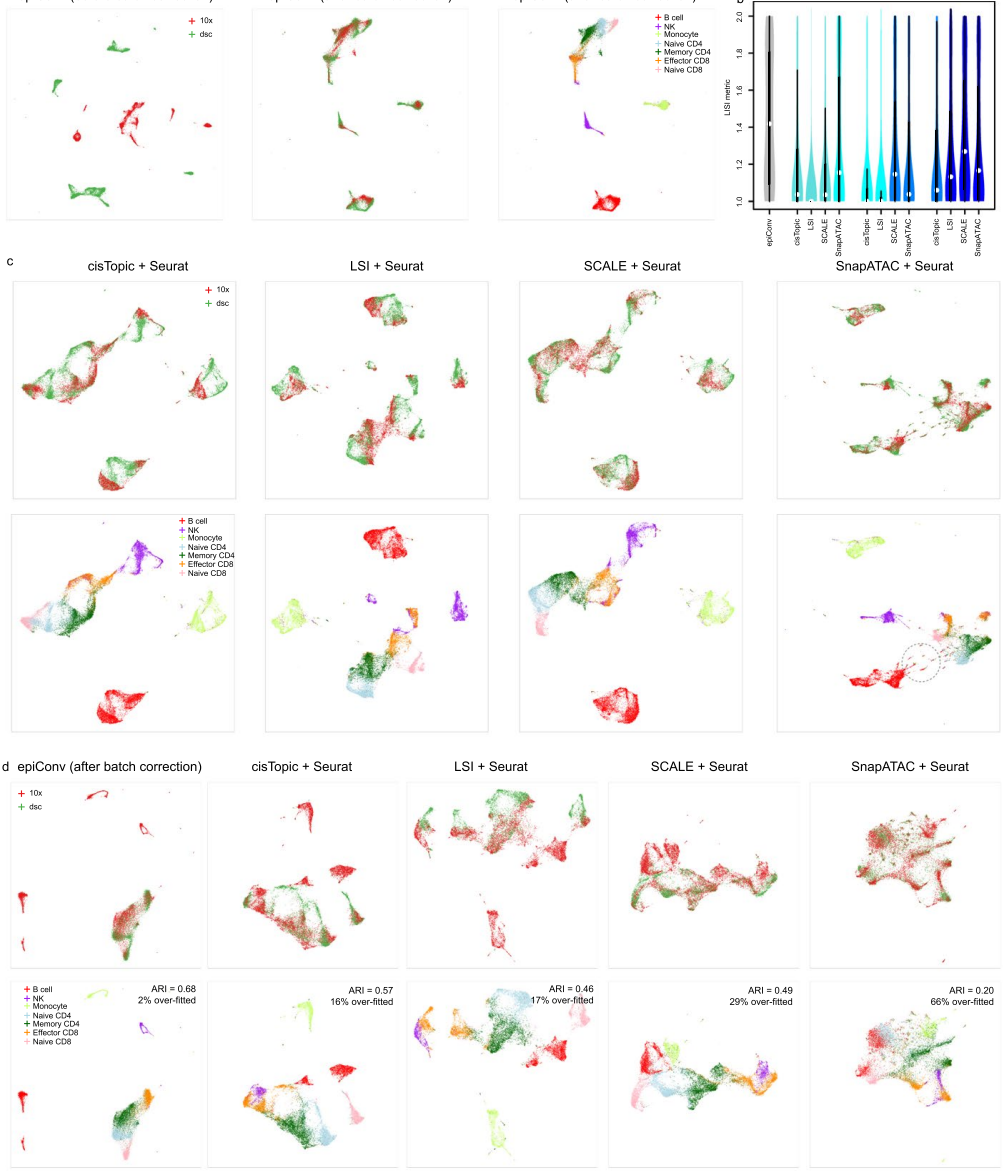
Benchmarking of epiConv on integrating two PBMC datasets. **a** Low dimensional embedding of PBMCs before and after batch correction. **b** local inverse Simpson’s Index (LISI) of different methods. The LISI is equal to the effective number of batches in each cell’s neighborhood. **c** Low dimensional embeddings of PBMCs after batch correction by Seurat based methods. **d** Comparison of epiConv and Seurat based methods after removing B cells, monocytes and NK cells from the reference (dsc).

We first applied epiConv to two peripheral blood mononuclear cells (PBMCs) datasets sequenced by two protocols (10x Genomics and dscATAC-seq) to compare its performance with other methods [5, 19]. The batch effects between two datasets were largely removed by epiConv (Fig. 2a). We used the LISI metric (equal to the effective number of batches in each cell’s local neighborhood) to quantitatively evaluate the mixing of cells from two batches and epiConv showed clear advantage over other methods (Fig. 2b). The UMP visualization also suggested that Harmony and MNN based methods failed to correct the batch effects while Seurat based methods performed better than them (Fig. 2c; Additional file 1: Fig. S2). Besides the batch correction, there were many small clusters with mixed cell identities in all SnapATAC results, suggesting technical biases derived from the step of latent feature learning but not batch correction (Fig. 2c; Additional file 1: Fig. S2; highlighted by dashed circle).

To perform further benchmarking between epiConv and Seurat (the one performed better among existing tools) based methods, we generated a new reference batch with only T cells and removed NK cells, B cells and monocytes from the reference (dscATAC-seq) and performed batch correction with the new reference again (Fig. 2d). EpiConv corrected the batch effects between T cells and grouped other cells as distinct clusters, suggesting that it was almost unaffected by the removal of NK cells, B cells and monocytes from reference. However, we found that some NK cells, B cells and monocytes in the query dataset were mixed with T cells in the reference dataset (the proportion of over-fitted cells in Fig. 2d; see Methods section for the definition of over-fitted cells) after Seurat batch correction, which resulted in lower clustering accuracy (ARI in Fig. 2d). The results of SCALE/SnapATAC + Seurat were severely affected as B cell and monocyte clusters were merged with T cells. The results of CisTopic/LSI + Seurat were less affected but there was still higher fraction of over-fitted cells than epiConv. These incorrectly placed cells were unlikely to be contaminations from experiments as they could not be found in Fig. 2c. We found that in the Canonical Correlation Analysis (CCA) space of Seurat when NK cells, B cells and monocytes were removed from reference dataset, T cells in the reference dataset became closer to other cell types in the query dataset (Additional file 1: Fig. S3a), which caused a large number of mis-identified anchors and subsequent over-fitting problem in batch correction (Additional file 1: Fig. S3b). These results suggested that Seurat based methods might be vulnerable to over-fitting in scATAC-seq data when certain cell types were uniquely present in query, but not in the reference dataset.

### EpiConv better retains the biological variations

**Fig. 3:**
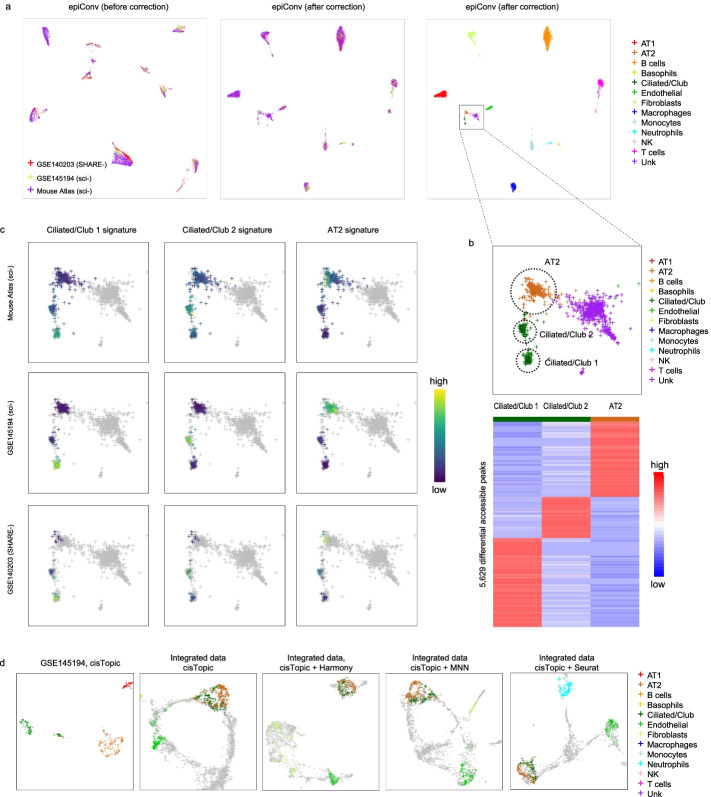
Benchmarking of epiConv on integrating three mouse lung datasets. **a** Low dimensional embedding of mouse lung data before and after batch correction. AT1, alveolar type 1 cell; AT2, alveolar type 2 cell. **b** EpiConv clusters AT2 cells and Ciliated/Club cells into three clusters, each with its unique accessible peaks. **c** Cell-type specific signatures of AT2, Ciliated/Club 1 and Ciliated/Club 2 cells. **d** CisTopic detects the difference between AT2 and Ciliated/Club cells on single dataset but failed on integrated datasets.

We also evaluated the performance of epiConv and other methods on another mouse lung data [7, 20, 21]. EpiConv removed the batch effects between three datasets (Fig. 3a). To assess the accuracy of clustering, we annotated clusters according to original article [21] and performed differential analysis on three datasets. The majority of cell-type specific markers were consistent across datasets (Additional file 1: Fig. S4a). For Basophils and Neutrophils whose markers could not be detected due to low abundance in some datasets, we performed differential analysis on cells of GSE145194 and calculated cell-type specific signatures for single cells. The results suggested that Basophils and Neutrophils were also correctly aligned (Additional file 1: Fig. S4b). Other methods also removed the batch effects between datasets but always mixed Ciliated/Club cells and AT2 cells together (Additional file 1: Fig. S5). Moreover, epiConv suggested that Ciliated/Club cells could be further segregated into two groups. To validate our findings, we performed differential analysis on three groups of cells (Ciliated/Club 1, Ciliated/Club 2 and AT2 cells) from GSE145194 to obtain their marker regions (Fig. 3b) and calculated the cluster-specific signatures across three datasets (Fig. 3c). The results suggested that such biological heterogeneities indeed existed as we could obtain a large number of markers for each cluster. Three types of cells were correctly grouped together by epiConv according to cell-type specific signatures. Although some methods could also segregate ciliated/club and AT2 cells, they could not segregate two ciliated/club subtypes (Additional file 1: Fig. S5).

We found that mixing of Ciliated/Club cells and AT2 cells was not caused by batch correction but by the upstream clustering algorithms. We used cisTopic as an example to show this phenomenon. We applied cisTopic to a single dataset (GSE145194) and found that cisTopic could also segregate AT2 cells from Ciliated/Club cells and detect two sub-clusters of Ciliated/Club cells (Fig. 3d). However, when applied to combined datasets, cisTopic failed to detect the difference between AT2 cells and Ciliated/Club cells even before batch correction. This result suggested that batch effect did not only introduce a systematic difference between cells of different batches but also blurred the biological signal in latent features. In such case, no batch correction method could segregate different types of cells when directly applied to latent features, as the information was already lost.

### Aligning co-assay data to scATAC-seq references

**Fig. 4:**
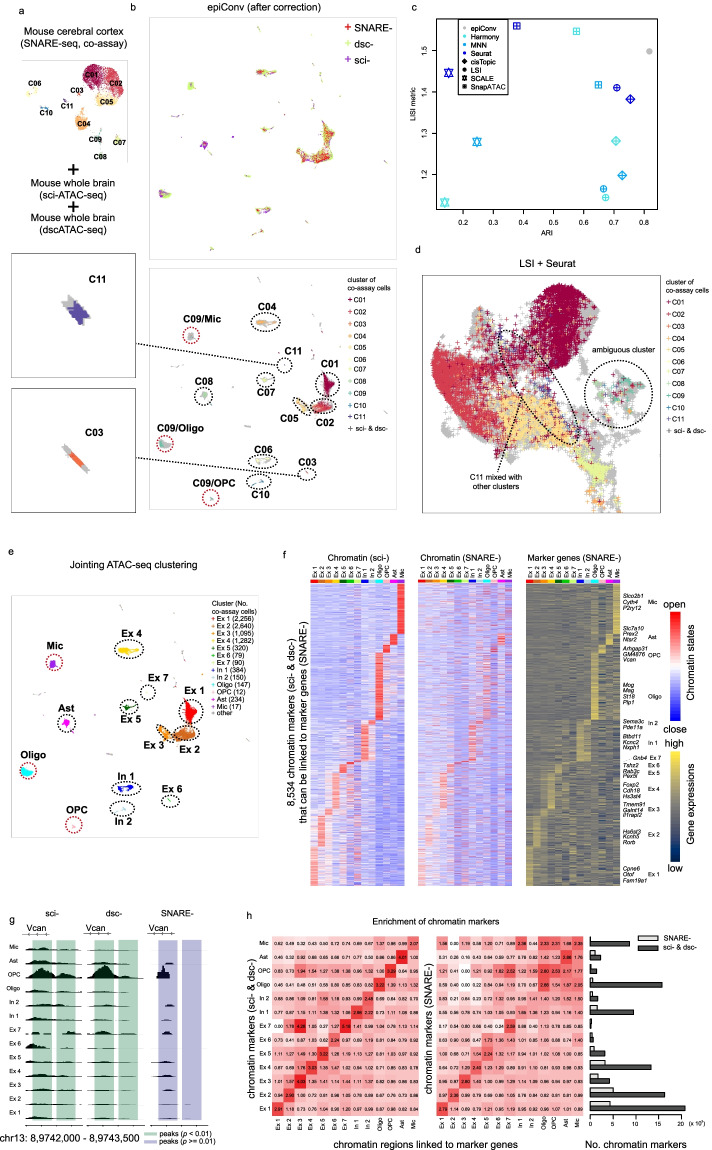
Aligning co-assay (SNARE-seq) data of mouse cerebral cortex to scATAC-seq (sci-ATAC-seq and dscATAC-seq) references increases the resolution of chromatin profiles. **a** Low dimensional embedding and clustering of co-assay data based on scRNA-seq profiles. **b** Joint embedding of scATAC-seq from co-assay data and scATAC-seq references. **c** The Adjusted Rank Index (ARI) and LISI of different methods. **d** Result of LSI + Seurat in details. LSI + Seurat groups cells with mixed identities together. **e** Joint embedding of scATAC-seq from co-assay data and scATAC-seq references, cells are colored by manually annotated joint clusters. Ex, excitatory neuron; In, inhibitory neuron; Oligo, oligodendrocyte; OPC, oligodendrocyte progenitor cell; Ast, astrocyte; Mic, microglia. **f** Heatmap of chromatin markers and their nearest genes. Left: chromatin profiles of sci-ATAC-seq. Middle: chromatin profiles of SNARE-seq. Right: gene expression profiles of SNARE-seq. Selected marker genes with highest fold changes are shown in the right panel. **g** Aggregated ATAC-seq signals near the TSS of OPC maker gene *Vcan*. **h** Left (scATAC-seq) and middle (co-assay): The fold changes of enrichment between ATAC-seq defined marker regions and RNA-seq defined associated regions. Right: the number of detected markers from scATAC-seq references and co-assay data for each cluster.

EpiConv can also increase the resolution of chromatin profiles through joint analysis. Next, we demonstrated that aligning the ATAC-seq profiles of co-assay data (perform scRNA-seq and scATAC-seq simultaneously on the same cells) onto scATAC-seq references overcame the shortcomings of shallow sequencing depth in co-assay data and improved the performance of clustering and differentially accessible peaks calling. We integrated three datasets together (Fig. 4a), one co-assay dataset of adult mouse cerebral cortex [6] (SNARE-seq) as query dataset, two scATAC-seq datasets of adult mouse brain [5, 7] (sci-ATAC-seq and dscATAC-seq) that served as references, whose sequencing depth were ~5 times deeper than the scATAC-seq of co-assay data. Single cells from three datasets were mixed together without obvious batch effects by epiConv and the embedding were consistent with RNA-seq derived clusters (Fig. 4b). Rare cell types (C03 and C11) were also clearly segregated by epiConv (Fig. 4b).

The low dimensional embeddings of other methods suggested that they could not fully remove the batch effects or were inconsistent with RNA-seq derived clusters (Additional file 1: Fig. S6). To quantitatively evaluate their performance, we used LISI metric to assess batch mixing and ARI to assess the consistency between clustering of ATAC-seq and RNA-seq. EpiConv outperformed most methods in both batch mixing and accuracy (Fig. 4c). The overall performance of LSI + Seurat is similar compared to epiConv. However, the clustering of ATAC-seq data by LSI + Seurat did not agree with RNA-seq profiles (Fig. 4d). RNA-seq defined rare excitatory neuron C11 were mixed with other excitatory neurons (C01, C02 and C05) and a small fraction of cells from various RNA-seq derived clusters formed single cluster in ATAC-seq data (the ambiguous cluster in Fig. 4d). SnapATAC + Harmony and SnapATAC + Seurat performed better in batch mixing than epiConv but with lower accuracy. The UMAP visualization showed that SnapATAC + Harmony grouped cells of C01, C02 and C05 into many small clusters and SnapATAC + Seurat grouped a large proportion of cells from C01, C02 and C05 into a single cluster, both of which showed strong contradictions with scRNA-seq data (Additional file 1: Fig. S6b).

Based on joint ATAC-seq clustering of integrated dataset (Fig. 4e, clusters were manually annotated by canonical makers), we found that RNA-seq derived cluster C09 could be further grouped into 3 cell types (Fig. 4e, clusters highlighted by red circles), which referred to one major cell type (oligodendrocyte) and two rare cell types (oligodendrocyte progenitor cell and microglia) based on their highly expressed genes, suggesting that joint analysis increased the resolution of clustering for rare cell types or subtypes.

Next, we examined whether single cells of co-assay data were correctly assigned to the references. In each dataset, we performed differential analysis to get a set of marker peaks for each joint cluster to see whether these markers were conserved across different datasets. Although markers detected from two scATAC-seq references were consistent with each other, a significant portion of these markers were not clearly more accessible in the corresponding cell types of co-assay data (Additional file 1: Fig. S7a). Only markers of clusters with a large number of cells were conserved across three datasets (e.g. Ex 1–4) and many cluster-specific markers could only be detected from deep sequencing references. We argued that this might be due to shallow sequencing of co-assay data and further incorporated RNA-seq profiles to demonstrate that these peaks inferred from matched high depth references could be supported by transcriptome data. First, we linked each peak to its closest promoter in the genome. Then for each cluster, we got a series of associated peaks with its cluster-specific marker genes. We found 8534 chromatin markers from scATAC-seq references that showed consistent pattern with its associated genes (Fig. 4f, left and right), revealing the potential relationships between cis-regulatory elements and their target genes. However, only a small fraction of these peaks (3031 peaks) was differentially accessible in SNARE-seq (Fig. 4f, middle). Other peaks were not statistically significant or completely undetectable due to shallow sequencing. For instance, the ATAC-seq peaks near the promoter of *Vcan*, an OPC marker suggested by RNA-seq data, didn’t pass statistical threshold before integrating references (Fig. 4g).

To quantitatively measure the consistency between ATAC-seq and RNA-seq profiles, we calculated the fold change of enrichment between chromatin markers and marker gene associated peaks for each cluster (*N*_*common*_/(*N*_*ATAC*_ * *N*_*RNA*_) * *N*_*total*_; *N*_*common*_: number of shared peaks, *N*_*ATAC*_: number of chromatin markers, *N*_*RNA*_: number of marker gene associated peaks, *N*_*total*_: number of total ATAC-seq peaks). We expected that peaks residing near the promoter of one gene were more likely to be its cis-regulatory elements and cluster-specific chromatin markers would enrich in the nearby regions of marker genes if joint analysis correctly clustered recurrent cell types from co-assay and scATAC-seq datasets together. Indeed, we observed the enrichment and for most clusters, we detected much more chromatin markers from scATAC-seq references and they were with similar or even higher extent of enrichment with marker gene associated peaks than chromatin profiles of SNARE-seq (Fig. 4h), proving that epiConv correctly assigned co-assay single cells to the references and increased sensitivity of differentially accessible peaks calling. We noted that there were few chromatin markers of Ex 6 and Ex 7 due to their small cluster size. In order to overcome this problem, we performed the analyses on 7 excitatory neuron clusters only and the results also supported the correctness of epiConv (Additional file 1: Fig. S7b).

### Aligning cells in different biological conditions

**Fig. 5:**
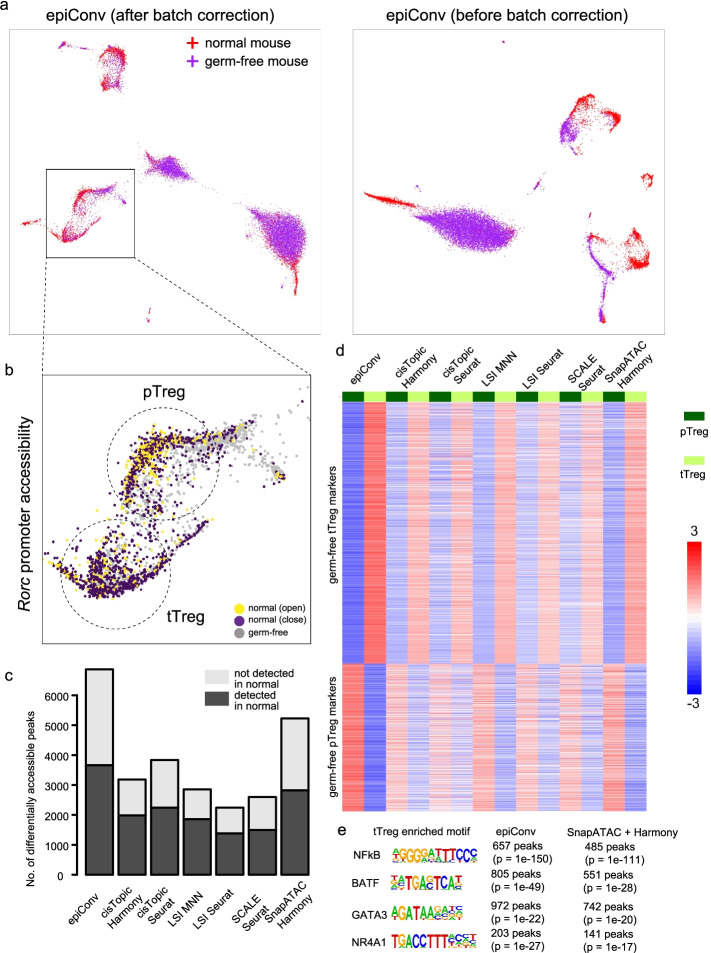
Integrative analysis based on EpiConv better detects tTreg signature in germ-free mouse. **a** Low dimensional embedding of epiConv before and after integration. **b** The accessibility of *Rorc* promoter in normal mouse shows the identities of thymic Tregs (tTregs, *Rorc*^*close*^) and peripheral Tregs (pTregs, *Rorc*^*open*^). **c** The number of differentially accessible peaks between tTregs and pTregs in germ-free condition. **d** The Z-score normalized values with corresponding mean and variance of hypergeometric distribution in differentially accessible peaks. EpiConv derived clusters show more significant enrichment of accessible peaks in tTreg and pTreg markers. **e** Enriched motifs in up-regulated peaks of tTregs defined by epiConv and SnapATAC + Harmony.

In addition to batch correction, integration algorithms could also be used to align cells in different biological conditions. Here, we benchmarked the performance of epiConv by integrating CD4+ T cells from normal and germ-free mouse colon [22]. In the original article, cells in normal and germ-free conditions were analyzed separately. Peripherally induced Treg cells (pTreg) were not found in germ-free condition but thymus-derived Treg cells (tTreg) still existed. Consistent with original article, we also found two distinct clusters referring to pTreg and tTreg in normal mouse but could only find one Treg cluster from germ-free mouse that matched the markers of tTreg (Additional file 1: Fig. S8a) when analyzing two datasets separately. However, when CD4+ T cells from the two conditions were integrated by epiConv, there were two groups of cells from germ-free mouse that were clustered with pTreg and tTreg of normal mouse, separately (Fig. 5a,b). To validate our findings, we performed differential analysis (tTreg vs. pTreg) on normal and germ-free mouse and found that there were a large proportion of conserved tTreg and pTreg markers in normal and germ-free conditions (Additional file 1: Fig. S8b), suggesting that Treg in germ-free condition could also be classified to two groups similarly but they could not be easily distinguished from each other without proper reference.

We also applied other methods for comparison. Six method combinations removed the difference between T cells in two conditions (cisTopic + Harmony, cisTopic + Seurat, LSI + MNN, LSI + Seurat, SCALE + Seurat, SnapATAC + Harmony) and showed similar results that Tregs in both conditions could be classified into two groups (Additional file 1: Fig. S8c). The differentially accessible peaks detected by epiConv and other six methods were highly consistent in both normal and germ-free conditions, suggesting that they all captured the variations between pTreg and tTreg. However, the number of differentially accessible peaks in germ-free condition detected by epiConv was much more than other methods (all methods detected close number of differentially accessible peaks in normal condition) and about half of them could also be detected in normal condition (Fig. 5c). As our differential analysis was based on hypergeometric test, clusters of higher purity would result in more differentially accessible peaks to be detected due to the enrichment of accessible peaks in one cluster. To assess the cluster purity of different methods, we normalized the differentially accessible peaks detected by epiConv and other six methods using the corresponding mean and variance of hypergeometric distribution. The clusters inferred by epiConv clearly better segregated accessible vs. inaccessible peaks and consequently resulted in more significant enrichment of accessible peaks in one cluster than other methods (Fig. 5d).

We performed motif calling on differentially accessible peaks inferred by epiConv and SnapATAC + Harmony (the method that detected the second most differentially accessible peaks) to see whether they were associated with some TFs or simply due to technical noise (Fig. 5e). In up-regulated peaks in tTreg, we found four enriched motifs in germ-free condition. From the result of SnapATAC + Harmony, we also found the same set of enriched motifs but there were always fewer peaks containing the motif, which resulted in lower significance level. Given that these four motifs could also be detected in normal condition (data not shown), these results suggested that epiConv better detected the unique biological features of tTreg in germ-free condition. The motif of *Rorc* could be detected from pTreg in normal condition as expected (data not shown) but not in germ-free condition. No other motifs could be detected for pTreg in both conditions. Thus, the lack of *Rorc* mediated open chromatin might weaken the difference between two types of Tregs in germ-free condition and make it hard to segregate them.

### Aligning leukemic cells to normal hematopoiesis

**Fig. 6:**
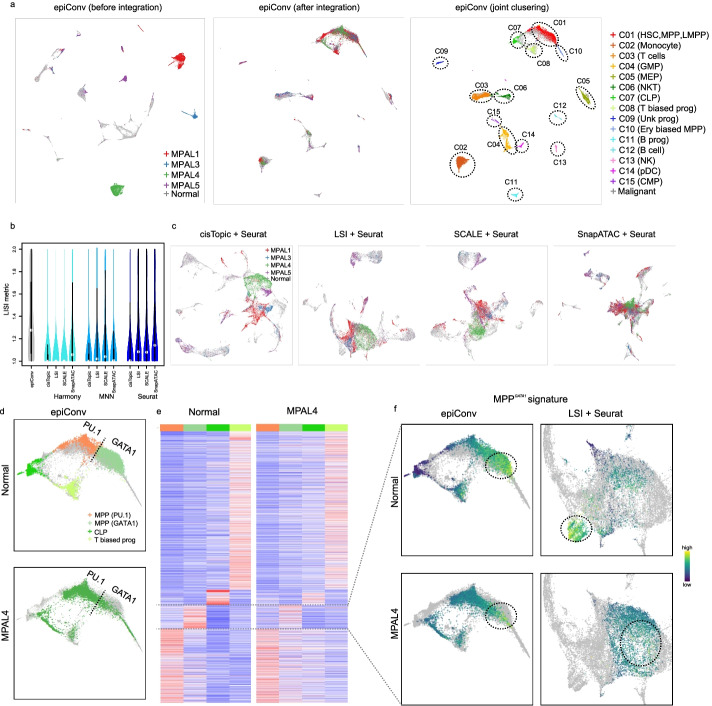
EpiConv better aligns leukemic cells to normal hematopoiesis **a** Low dimensional embedding of epiConv before and after integration. HSC, hematopoietic stem cell; MPP, multipotent progenitors; LMPP lymphoid-primed multipotent progenitor; GMP, granulocyte-macrophage progenitor; MEP, megakaryocyte-erythroid progenitor; CLP, common lymphoid progenitor; Ery biased MPP, erythroid biased MPP; pDC, plasmacytoid dendritic cell; CMP, common myeloid progenitor, T biased prog, T cell biased progenitor; Unk prog, unknown progenitor. **b** The LISI metric of different methods. **c** Low dimensional embeddings of cisTopic, LSI, SCALE and SnapATAC after Seurat batch correction. **d** Sub-clusters of C01 with increased accessibility in PU.1 or GATA1 motifs and two lymphoid primed clusters (CLP and T biased prog). Malignant cells of MPAL4 projected to these four clusters were shown. **e** Cluster-specific markers shared by normal cells and malignant cells of MPAL4. **f** MPP^*GATA1*^ signatures of normal cells and MPAL4 on the embedding of epiConv and LSI + Seurat.

We further benchmarked the performance of epiConv on a leukemia dataset by aligning malignant cells from mixed-phenotype acute leukemia (MPAL), which was known to present with features of multiple hematopoietic lineages, to normal hematopoiesis [23]. The normal hematopoiesis reference contains bone marrow mononuclear cells (BMMCs) and CD34+ enriched BMMCs and the Leukemia data contains single cells from MPAL patients [23]. After epiConv integration, all malignant cells were projected to the hierarchy of normal hematopoiesis (Fig. 6a). Most method combinations failed to project the malignant cells to normal hematopoiesis (Additional file 1: Fig. S9a,b). Seurat based methods performed better than others but still worse than epiConv (LISI metric, Fig. 6b; embeddings, Fig. 6c). We annotated clusters by comparing chromatin markers of normal cells with FACS-sorted bulk samples [24] (Fig. 6a right; Additional file 1: Fig. S10a). In addition to known cell types, we also found two novel clusters (C08 and C09). The C08 cluster (T biased progenitor) was similar to stem cell cluster (C01) but was more accessible in the marker regions of T cells (~ 50% up-regulated peaks in C08 against C01 are T cell markers) and the C09 cluster (unknown progenitor) was moderately accessible in myeloid, lymphoid and erythroid lineage-specific regions. All malignant samples contained a large proportion of stem-like cells in C01 (from 27.5 to 63.7%, Additional file 1: Fig. S10b, bottom line). Except for some clusters with few cells, malignant cluster-specific markers always showed the highest fold change of enrichment with corresponding markers of normal cells, suggesting that leukemic cells were assigned to the most similar normal cells (Additional file 1: Fig. S10b).

As the hematopoiesis dataset was the most strongly affected dataset by residuals of Eigenvalue decomposition, we also used it as an example to show how the residuals helped correct batch effects. Through UMAP embedding and LISI metric, we found that the batch effects in the corrected Eigenvector space without residuals were reduced but not fully corrected (Additional file 1: Fig. S9c,d). Thus, the results proved that the residuals of Eigenvalue decomposition contained biological signals that were essential to recover shared identities of cells from different conditions.

Given that hematopoietic stem and progenitors cells (C01) contained multipotent progenitors with different lineage priming, we were interested in whether similar lineage bias could also be observed in malignant cells. To better reveal the lineage priming of cells in C01, we grouped C01 to two sub-clusters, one cluster with increased accessibility in PU.1 motifs (MPP^*PU.1*^) and the other with increased accessibility in GATA1 motifs (MPP^*GATA1*^) in normal cells, reflecting bias of MPPs to myeloid and erythroid lineages (Fig. 6d). We performed differential analysis on normal and malignant cells in MPP^*PU.1*^, MPP^*GATA1*^ and two lymphoid priming clusters (CLP/C07 and T biased prog/C08) to find conserved markers between normal and malignant cells, which could directly support our assignments of malignant cells. As expected, the corresponding cluster-specific markers between normal and malignant samples always showed higher fold change of enrichment (Additional file 1: Fig. S11a). Malignant cells of MPAL1, MPAL4 were projected to all of the four clusters and shared a common set of 3,892 conserved markers with normal cells (MPAL4, Fig. 6e; MPAL1, Additional file 1: Fig. S11b). Most cells of MPAL3 were projected to MPP^*GATA1*^ and T biased prog (C08) and also agreed with MPAL1 and MPAL4 and shared the same set of conserved markers (Additional file 1: Fig. S11b, clusters < 250 cells were not included in differential analysis). Malignant cells of MPAL5 were mainly projected to MPP^*PU.1*^ and T biased prog (C08). A large number of conserved markers between MPAL5 and normal cells could still be found but they differed from the conserved markers of other malignant samples (Additional file 1: Fig. S11c).

As cluster-level analysis may miss intra-cluster heterogeneities, we calculated the cluster-specific signatures of cells based on the conserved markers detected above to investigate their lineage bias in single cell level (MPP^*GATA1*^ signature of normal cells and MPAL4 shown in Fig. 6f; other signatures shown in Additional file 1: Fig. S11d). The results also agreed with cluster-level analysis that normal and malignant cells grouped together according to their lineage bias, but the signal sometimes was much weaker in malignant cells (e.g. CLP signature in MPAL4, Additional file 1: Fig. S11d). Additionally, a synchronized decrease of MPP^*PU.1*^ signature and increase of MPP^*GATA1*^ signature from MPP^*PU.1*^ to MPP^*GATA1*^ could be observed in both normal and malignant cells (MPAL1 and MPAL4, Additional file 1: Fig. S11d), suggesting that epiConv corrected aligned the continuum of lineage bias transition between MPP^*PU.1*^ and MPP^*GATA1*^.

Using the signatures calculated above, we next examined whether leukemic cells were also correctly projected to their normal counterparts by LSI + Seurat. Although some malignant cells in MPAL1, MPAL3 and MPAL4 were indeed with higher erythroid potential, LSI + Seurat did not project them to the corresponding normal cells (Fig. 6f; cells with higher MPP^*GATA1*^ signature were grouped together in epiConv but no in LSI + Seurat). In fact, no malignant cells were projected to MPP^*GATA1*^ normal cells. These results suggested that epiConv better integrated normal and malignant cells according to their lineage bias than LSI + Seurat. We also examine the lineage priming of SCALE/SnapATAC + Seurat, which showed higher LISI metric than other methods and they also could not integrate normal and malignant cells together according to their lineage bias (Additional file 1: Fig. S12).

### Computational efficiency of epiConv

We finally benchmarked the computational efficiency of epiConv on a human bone marrow and blood mononuclear cells dataset of 100k cells. We down-sampled the dataset to 10k, 20k, 50k and 100k cells and applied epiConv on them. EpiConv could run in parallel, achieved desirable results and was computationally efficient on large datasets (~4 h running time with 5 threads for dataset of 100 k cells, Additional file 1: Fig. S13). LSI and SnapATAC showed similar computational efficiency with epiConv. However, SnapATAC run out of memory on dataset of 100k cells (require more than 78 GiB memory). CisTopic and SCALE were less efficiency and required more than 24 hours for large dataset (cisTopic, ≥ 20 k cells; SCALE, ≥ 100 k cells).

## Discussion

In this paper, we introduced a novel method named epiConv for joint analysis of scATAC-seq data and compared it with several clustering tools of scATAC-seq combined with batch correction tools designed for scRNA-seq. When applied to scATAC-seq data, these batch correction tools generally did not match their performance in scRNA-seq data. We found that batch effects and biological space were not always orthogonal to each other in scATAC-seq data (Additional file 1: Fig. S3a), which broke the assumption of existing batch correction tools [14, 17] and caused over-fitting problem (Fig. 2d). Moreover, technical bias might be introduced by clustering tools of scATAC-seq data during the step of latent feature learning and was inherited by batch correction tools (Fig. 3d). To solve this problem, the potential batch effects between datasets should be considered in latent feature learning, but unfortunately such functionality is not available in state-of-art scATAC-Seq tools. Therefore, a one-stop working pipeline, such as epiConv, that performs clustering and batch correction simultaneously should handle the problem better in joint analysis of scATAC-seq data.

The other unique feature of epiConv is the inclusion of residuals after dimension reduction. We found that most of existing integration algorithms failed to align cells from the different conditions (tumor vs normal) together (Fig. 6f). We hypothesized that the discarded residuals might contain biological information. Therefore, we compared the results of epiConv under two strategies (with or without adding back residuals), we found that including the residuals of Eigenvalue decomposition significantly improved the results of batch correction (Additional file 1: Fig. S9c,d). With this additional feature, we demonstrated that epiConv could also perform integration for datasets under different biological conditions (Fig. 6f). These results suggested that the residuals of dimension reduction might contain useful information in recovering the biological relationships between cells and should not be ignored.

Although epiConv cannot integrate scRNA-seq and scATAC-seq data directly, it is a useful tool to recover the promoter-enhancer relationships by improving the resolution of low-depth scATAC-seq of co-assay data. The low-depth of scATAC-seq limited the detection sensitivity of cell type specific chromatin profiles, especially for the rare cell population. We demonstrated that integration of scATAC-seq data with co-assay data linked a large number of enhancers to their putative target genes that could not be inferred from the co-assay data alone, while most of other methods failed to do so (Fig. 4b,d). Another alternative to infer promoter-enhancer relationships is to perform scATAC-Seq and scRNA-Seq separately from the same population and to integrate these two types of data by integration algorithm, such as Seurat [17]. Most algorithms that directly integrate scRNA-seq and scATAC-seq data first transform the epigenomic data into a gene activity matrix by summing the ATAC-seq counts around genes’ TSS or across the gene body. However, recent study suggested that gene activity matrix performed poorly on clustering of scATAC-seq data [10], while distal enhancers better characterized the cell identities [25]. Thus, it remains unclear whether these methods can provide high-resolution integration, especially for closely-related cell types. On the contrary, integration of high-depth independent scATAC-seq data with scATAC-seq from co-assay data circumvents the difficulty in matching cells from two separate assays and significantly improves the resolution of cell-type specific chromatin marks (Fig. 4f). We believe that such strategy will help resolve the landscape of enhancer-promoter interactions for rare or closely-related cell types when co-assay data is available.

## Conclusion

In this paper, we developed an algorithm named epiConv to integrate multiple scATAC-seq datasets. We have demonstrated that epiConv can better remove batch effects and retain biological variations than existing methods under various situations. Moreover, joint analysis provides deeper insights into the epigenetic regulation of single cells of different developmental stages as well as disease conditions. We believe that the computational framework in this study along with technical improvements could facilitate better interpretation of the roles of chromatin accessibility in epigenetic regulation of cells in the future.

## Methods

### Peak calling and matrix counting

We used peaks defined in original article or called peaks from two ends of fragments by MACS2 [26] (--nomodel --nolambda --keep-dup all --shift -95 --extsize 200) if processed peak file was unavailable. We counted the ends of fragments against peaks to obtain the count matrix. The count matrix was binarized. Cells with less than 1,000 accessible peaks were filtered out.

### Application and evaluation of other methods

We applied cisTopic [11], Latent Sematic Indexing [4] (LSI), SCALE [15] and SnapATAC [16] to scATAC-seq data and used Harmony [12], MNN [14] and Seurat [17] to remove batch effects. In cisTopic, the number of meta features (topics) was set to 20, 30, 40 and 50 and automatically determined. In other methods, the number of meta features was set to 50. In LSI and SCALE, we filtered peaks that were accessible in less than 1% cells following the tutorial or default settings. SnapATAC was performed on ATAC-seq fragments following the tutorials.

We applied Harmony, MNN and Seurat on meta features calculated above to correct batch effects. We did not directly apply Harmony, MNN or Seurat on the raw matrix or gene activity matrix that summed the reads near each gene’s TSS as these simple methods generally did not perform well [10]. MNN and Seurat require specification of reference batches. It was the same as epiConv and was reported below. Other unmentioned settings were all set to defaults.

We used local inverse Simpson’s Index (LISI) to quantitatively evaluate the mixing of cells from different batches by R package lisi [12].

### Infer differentially accessible peaks and cluster-specific signatures

We used hypergeometric test to detect cluster-specific accessible peaks. Population size was defined as total number of cells. Sample size was defined as the total library size of cluster divided by the mean library size of all cells. No. of success in the population for each peak was defined as total number of cells with coverage. No. of success in the sample was defined as number of cells with coverage in this cluster. Only peaks that were accessible in at least 1% cells were tested. Peaks with one-tailed *p *value smaller than 0.01 were considered as differentially accessible peaks. If peaks were considered as differentially accessible peak in more than one cluster based on the statistical significance threshold, we assigned the peak to the cluster with the highest normalized counts. Cluster-specific signatures of single cells were calculated by the total number of accessible cluster-specific markers divided by the total number of accessible peaks.

### scATAC-seq data of PBMCs

Basic data processing was described as above. Cells from GSE123581 were used as reference. As the exact identities of T cells were unknown, we used a supervised approach to annotate T cells. We first grouped T cells into 4 clusters (Naïve CD4+ T cells, Naïve CD8+ T cells, memory CD4+ T cells and effector CD8+ T cells) by epiConv and performed differential analysis to obtain the cluster-specific markers. We aggregated the cluster-specific markers to calculate cluster-specific signatures for each single cell. Finally, we normalized signatures by ${x}_{norm}= \frac{x-{x}_{0.01}}{{x}_{0.99}-{x}_{0.01}}$, where *x*_0.01_ and *x*_0*.*99_ is the 1% and 99% percentiles of the signature values across all cells, and annotated single cells by the highest normalized signature. Although epiConv was not guaranteed to be with the highest accuracy, the cluster-specific signatures should be weekly affected as far as it was with reasonable accuracy. We did not find strong contradictions between the annotations and the results of other methods. The identities of other cells (NK, monocytes and B cells) were obtained from original articles.

In over-fitting test, we used Adjusted Rand Index (ARI) to evaluate the consistency between clusters and annotated cell identities. As the resolution of Louvain clustering affected ARI, we performed Louvain clustering with resolution from 0.2 to 2.0 and reported the highest ARI for each method. To count the number of over-fitted cells, we classified single cells into T cells and non-T cells and calculated the LISI by this definition. Cells with LISI > 1.1 (which meant that T cells were mixed with non-T cells) were counted as over-fitted.

### scATAC-seq data of mouse lung data

Basic data processing, differential analysis and calculation of cell-type specific signatures were performed as described above. Cells from Mouse Cell Atlas were used as reference. SHARE-seq (GSE140203) is a multi-omics protocol that generates RNA-seq and ATAC-seq profiles simultaneously. But only a small proportion of cells (~ 10%) from GSE140203 have RNA-seq profiles and the sequencing depth is shallow. We did not use the data from RNA-seq profiles.

### scATAC-seq data of adult mouse brain

We integrated co-assay data of mouse adult cerebral cortex from GSE126074 [6] (SNARE-seq), scATAC-seq of adult mouse brain from Mouse Cell Atlas [7] (sci-ATAC-seq) and GSE123581 [5] (dscATAC-seq) together. For RNA-seq profiles of co-assay data, we used the pipeline of Seurat with default settings (find 2000 most variable genes and obtain 50 PCs from them) to perform dimension reduction, clustering and finding cluster-specific marker genes. In ATAC-seq analyses, we first aligned dscATAC-seq data to sci-ATAC-seq data and then aligned co-assay data to them. Given that the quality of RNA-seq is much better than ATAC-seq, we calculated the correction vector of non-anchor cells from anchor cells using the SNN graph from RNA-seq data instead. Dimension reduction and joint ATAC-seq clustering were performed on integrated dataset as described above. Joint ATAC-seq clusters that contained fewer than 10 co-assays cells were not included in downstream analysis.

We used Adjusted Rand Index (ARI) to evaluate the consistency between clusters from RNA-seq and ATAC-seq for epiConv and other methods. We performed Louvain clustering on other methods with resolution from 0.2 to 2.0 and reported the highest ARI for each method.

The cluster-specific markers shared by sci-ATAC-seq and dscATAC-seq were shown and used to infer the relationships between ATAC-seq and RNA-seq. We also performed differential analysis on excitatory neuron cells instead of all cells to better detect the cluster-specific markers of rare cell types (Ex 6 and Ex 7).

### scATAC-seq data of mouse CD4+ T cells

Basic data processing was described as above. Cells from normal condition were used as reference. We performed Louvain clustering on integrated datasets and manually annotated pTreg and tTreg clusters by the accessibility of four marker gene promoters (*Foxp3, Klrg1, Rorc, Ikzf2*) used in original article. Differentially accessible peaks calling between pTreg and tTreg of normal and germ-free mouse was performed as described above. Motif calling was performed by Homer [27] on the ± 200 bp regions from peak centers. The length of motif was set to 8, 10 and 12. All ATAC-seq peaks were used as background.

### scATAC-seq data of leukemia

Basic data processing was described as above. Normal cells were used as reference. There were other two samples (MPAL2 and MPAL5 relapse) in Granja et al. [23]. Although we could align them to normal hematopoiesis, they showed week correlations with normal hematopoiesis and could not be used for benchmarking. Thus, we did not show the results of them.

### scATAC-seq data of human bone marrow and blood mononuclear cells

We combined data from human bone marrow and blood mononuclear cells from GSE129785, GSE139369 and 10x Genomics demonstration data together. Data of GSE139369 was used as reference. We performed Louvain clustering on data after integration and manually annotated each cluster by cells with known identities from original article or analyses described above.

## Supplementary Information

**Additional file 1.** Figures S1 to S13, supplementary table and supplementary note.

## Data Availability

All data analyzed in this article are available in public databases and are summarized in Additional file 1: Table S1. EpiConv is available in Github (https://github.com/LiLin-biosoft/epiConv).
