# Supplementary material for: Joint analysis of scATAC-seq datasets using epiConv
Source: BMC Bioinformatics. 2022 Jul 29;23:309. doi: 10.1186/s12859-022-04858-w (PMC9338487; doi:10.1186/s12859-022-04858-w)
Supplement: Supplementary file 1 — Additional file 1. Figures S1 to S13, supplementary table and supplementary note. [file 12859_2022_4858_MOESM1_ESM.pdf]

# Supplementary material for "Joint analysis of scATAC-seq datasets using epiConv"

# Supplementary Table

**Table S1** Published data used in this study

| dataset | No. cells after QC | Source |
|---|---|---|
| Cell lines | 1174 | GSE65360 |
| PBMCs | 31122 | GSE129785 |
| | | GSE123581 |
| Mouse lung | 15337 | http://atlas.gs.washington.edu/mouse-atac |
| | | GSE145194 |
| | | GSE140203 |
| Adult mouse brain | 53762 | GSE126074 |
| | | GSE123581 |
| | | http://atlas.gs.washington.edu/mouse-atac |
| Mouse CD4+ T cells | 20522 | GSE156112 |
| Leukemia | 46510 | GSE139369 |
| Human bone marrow and | 102325 | GSE129785 |
| blood mononuclear cells | | GSE139369 |
| | | https://www.10xgenomics.com/resources/datasets |

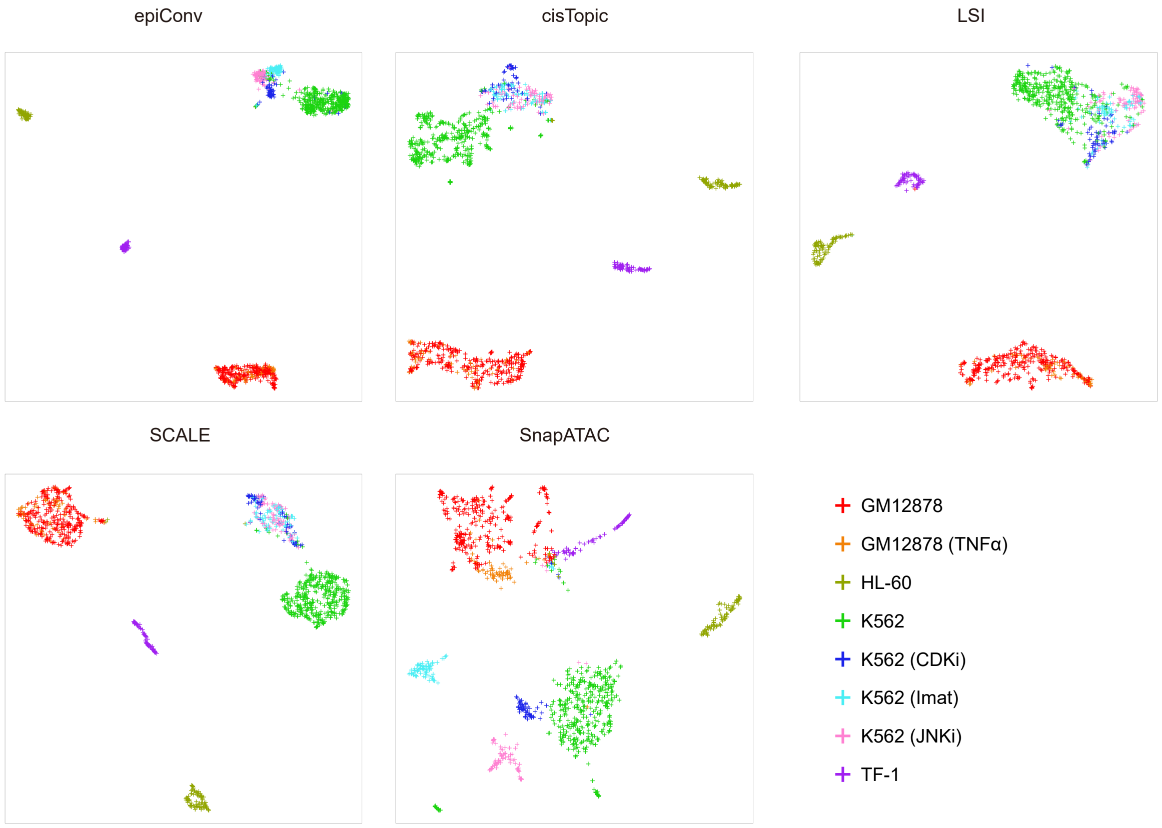


**Figure S1** Comparison of epiConv with cisTopic, LSI, SCALE and SnapATAC on cell lines data. Cells are colored by their identities.

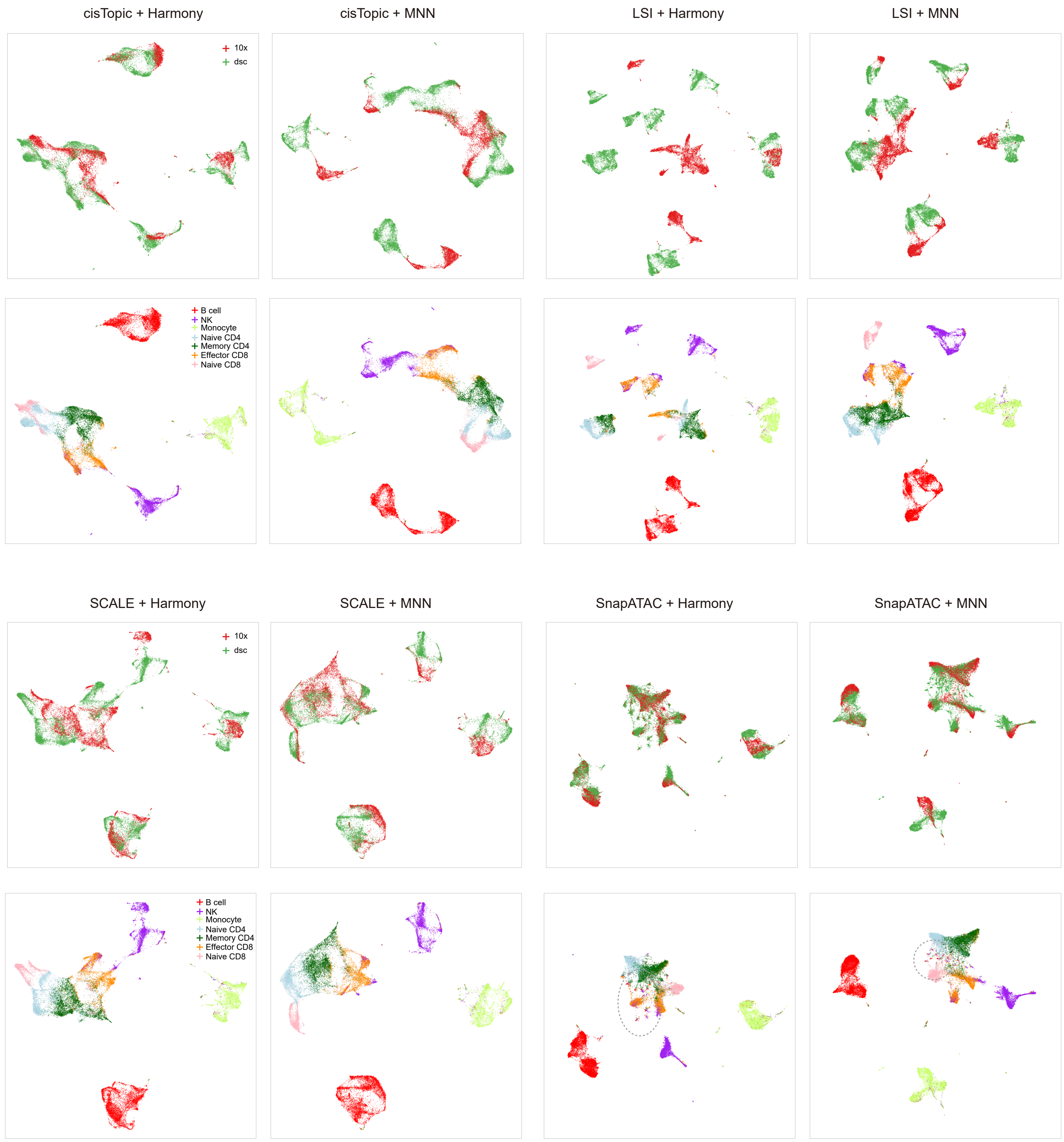


**Figure S2** Low dimensional embeddings of cisTopic, LSI, SCALE and SnapATAC after Harmony and MNN batch correction. The top panel is colored by the batch and the bottom panel is colored by cell identities. Dashed circles in SnapATAC based methods show clusters with mixed cell identities.

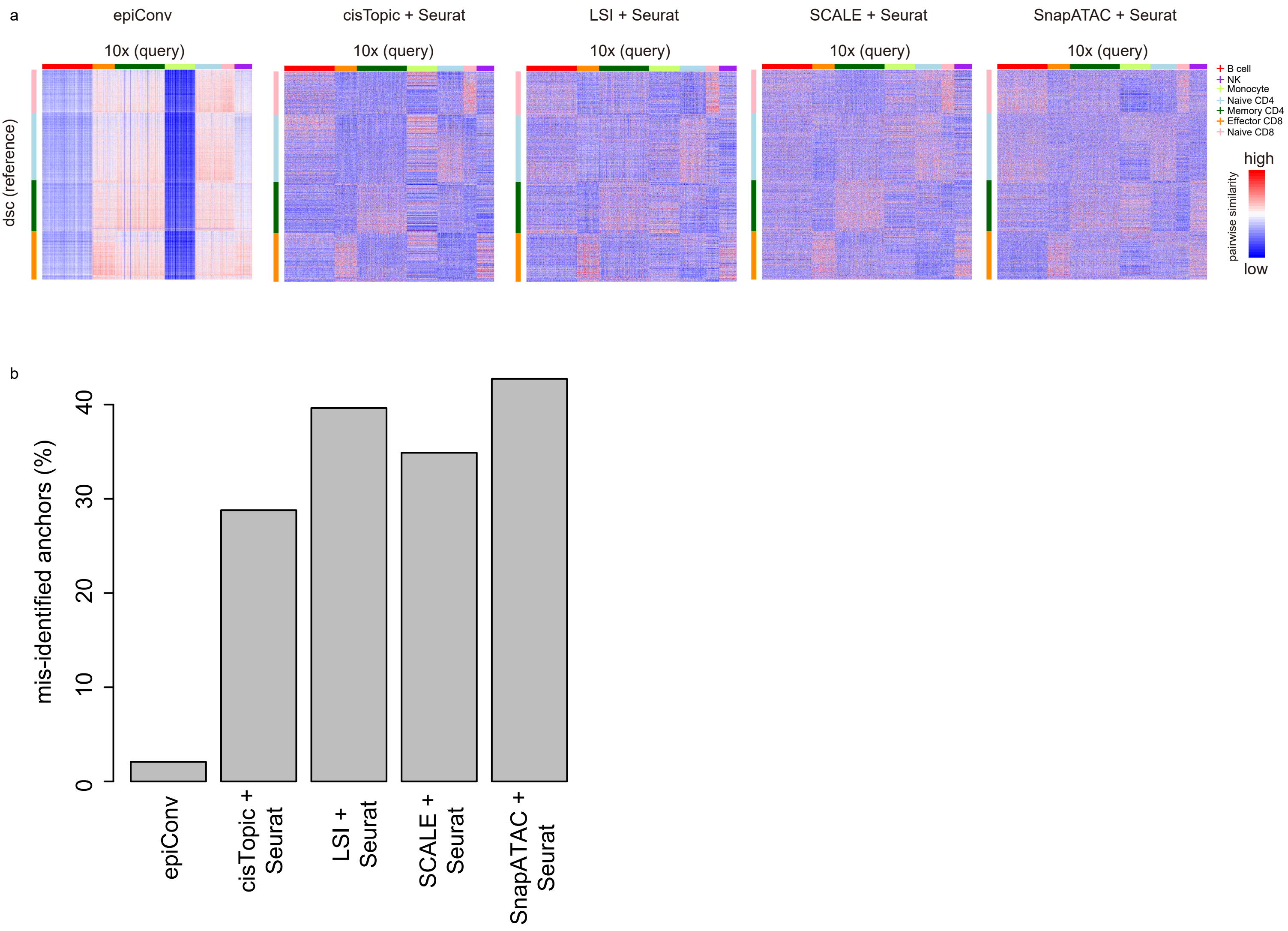


**Figure S3** Supplementary figures for **Fig. 2. a** The distances between cells from query and reference dataset in epiConv and Seurat based methods. **b** Number of mis-identified anchors (B cells, NK cells or monocytes in query dataset linkedto T cells in reference dataset) in epiConv and Seurat based methods.

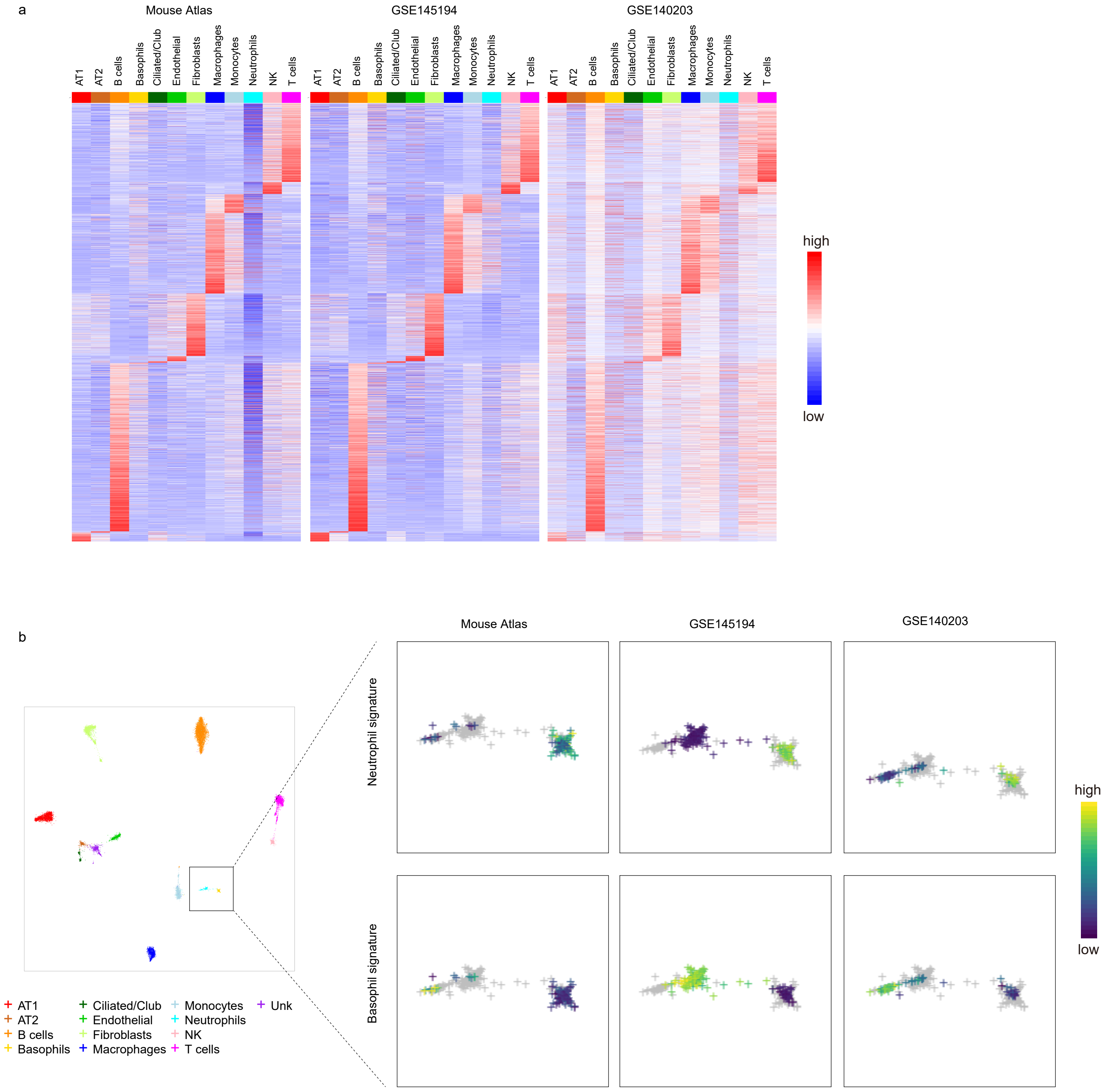


**Figure S4** Supplementary figures for **Fig. 3. a** Heatmaps of cell-type-specific chromatin markers shared by Mouse Cell Atlas and GSE145194. **b** Basophil and Neutrophil signatures across three datasets.

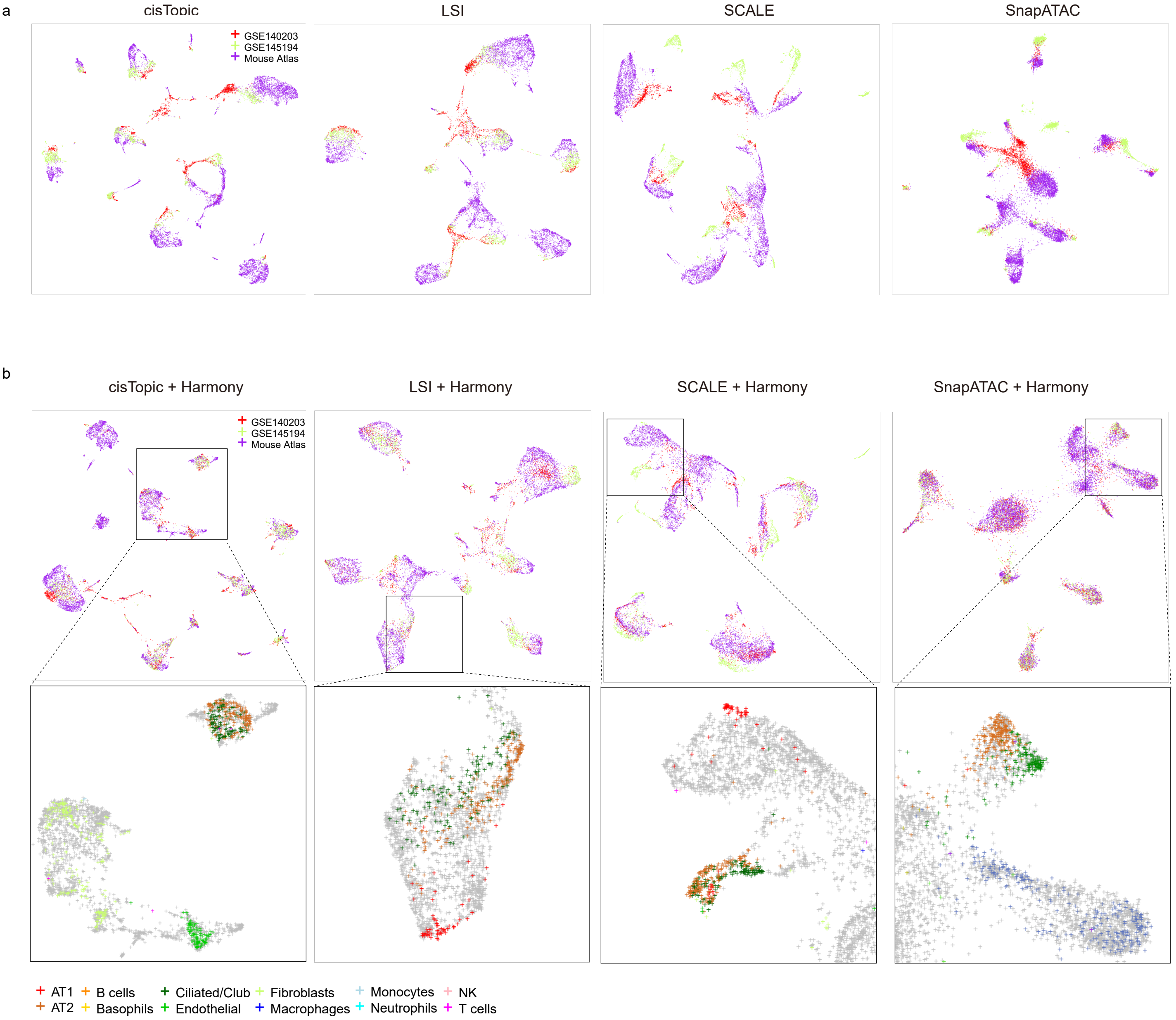


**Figure S5** Supplementary figures for **Fig. 3.** low dimensional embeddings of cisTopic, LSI, SCALE and SnapATAC before (**a**) and after (**b**) batch correction on mouse lung datasets. In (**b**), the top panel is colored by the batch and the bottom panel shows AT2 and Ciliated/Club cells annotated by original article.

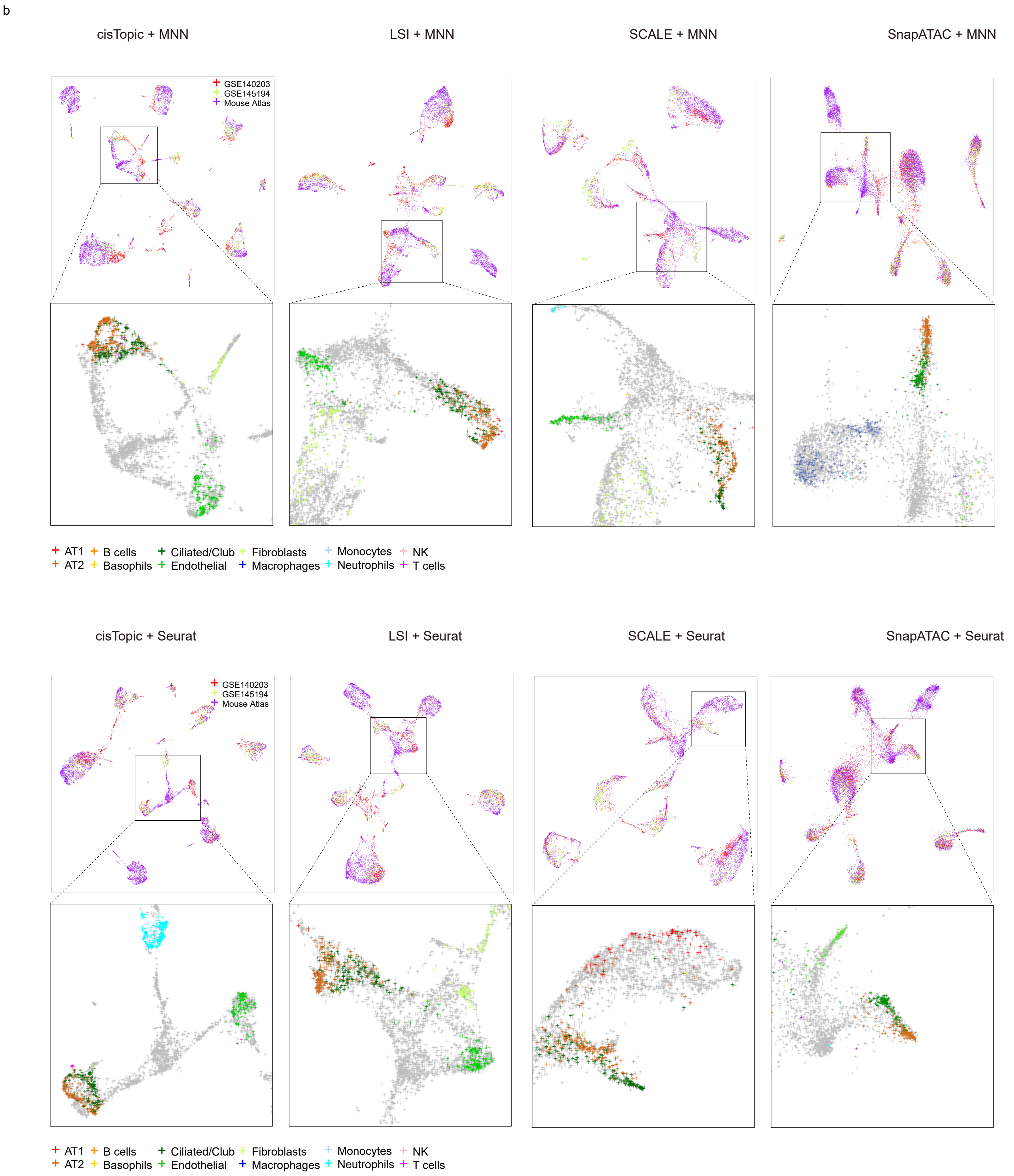


**Figure S5 continued**

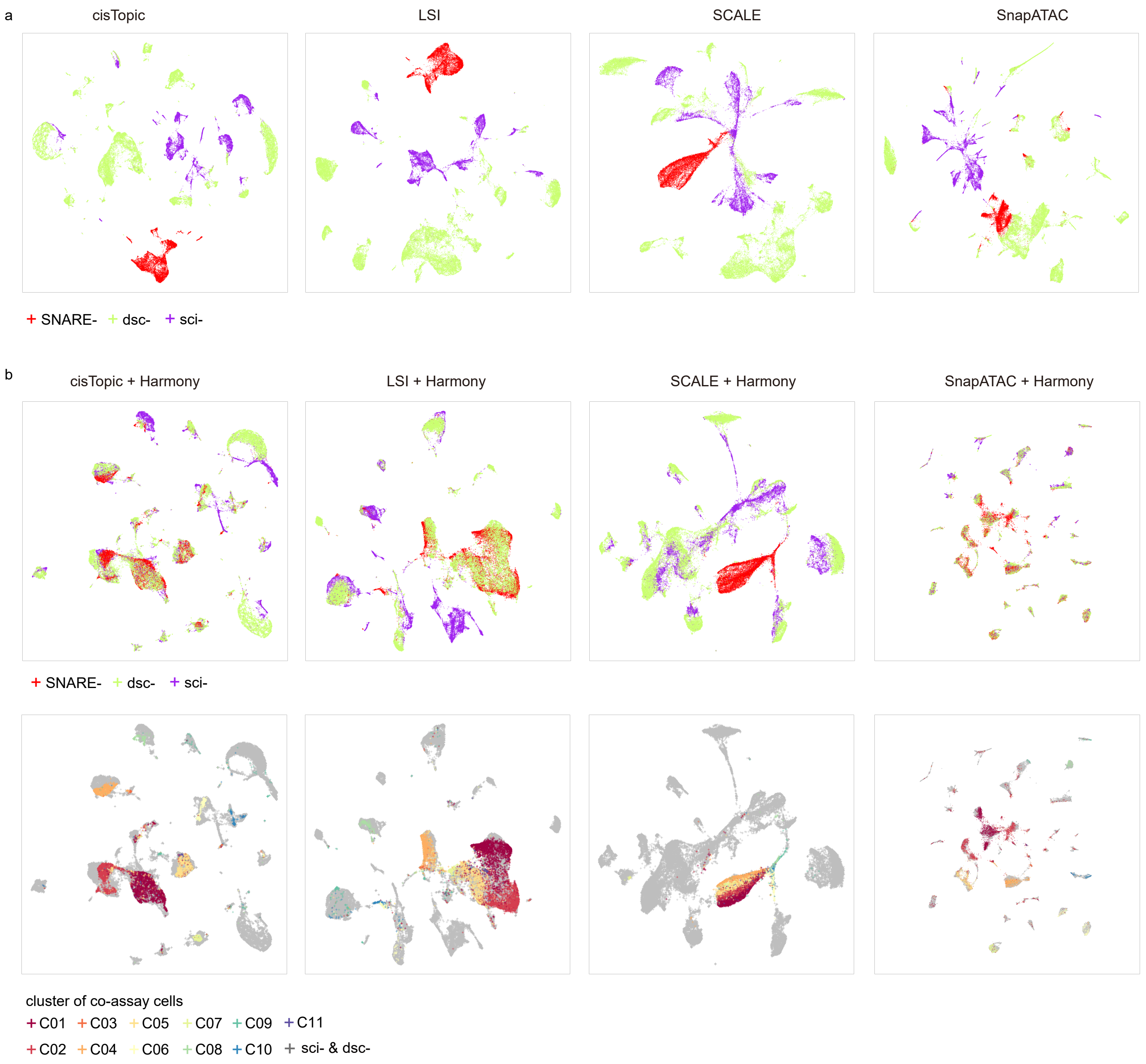


**Figure S6** Supplementary figures for **Fig. 4**. Low dimensional embedding of cisTopic, LSI, SCALE and SnapATAC before (**a**) and after (**b**) batch correction on adult mouse brain datasets. In (**b**), the top panel is colored by the batch and the bottom panel is colored by the RNA-seq derived clusters.

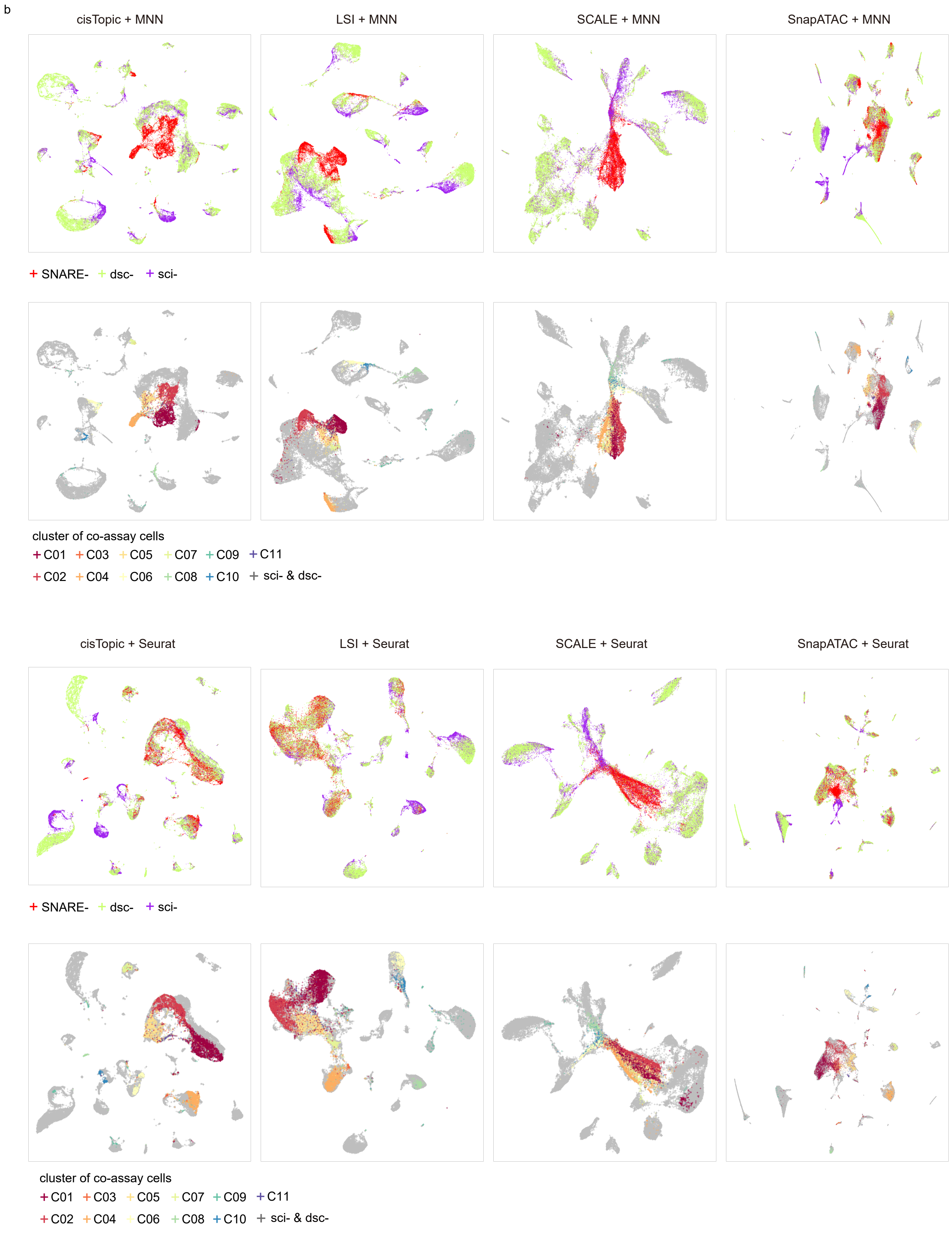


**Figure S6 continued**

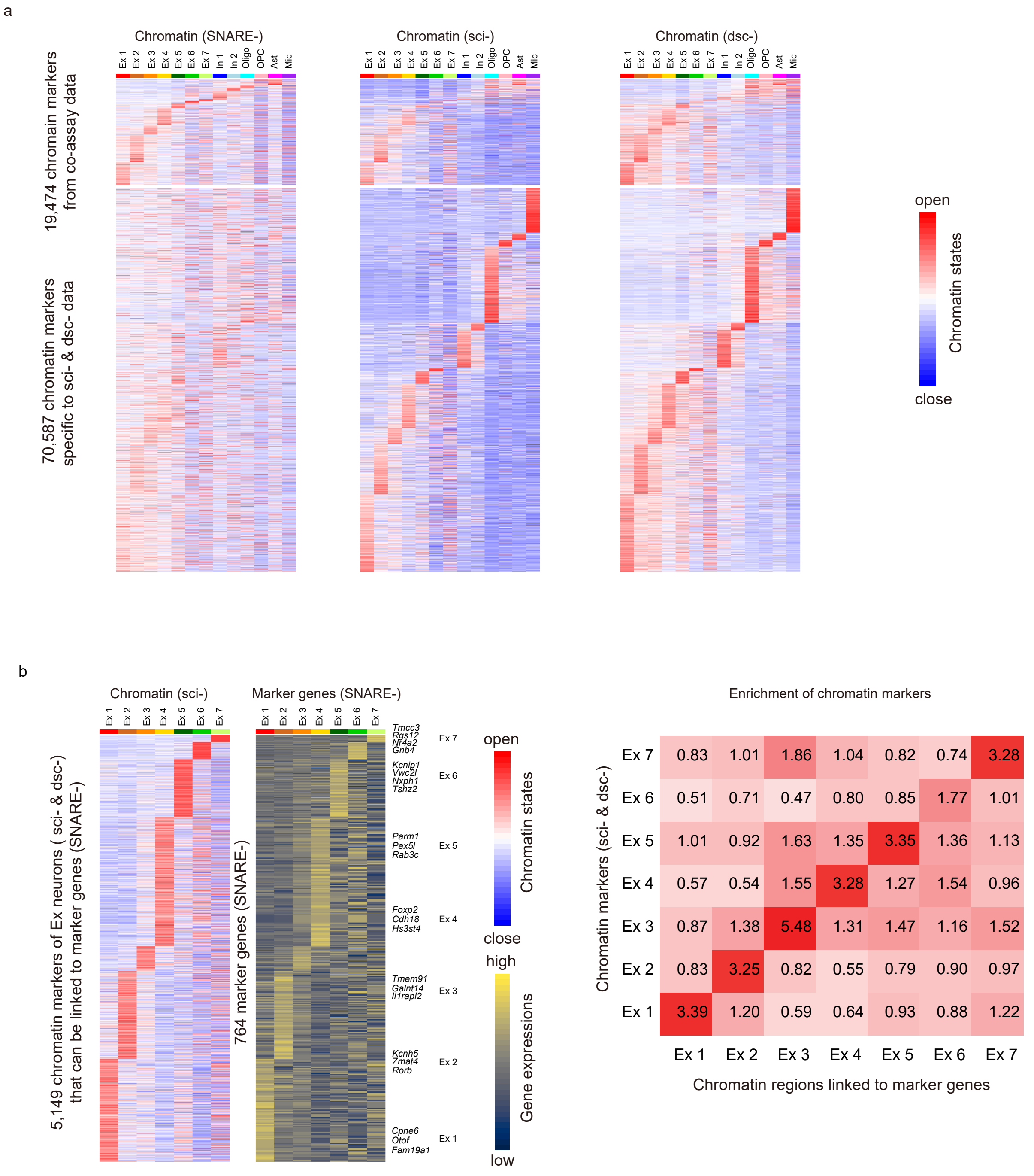


**Figure S7** Supplementary figures for **Fig. 4**. **a** Heatmaps of cluster-specific marker regions detected from co-assay data and two scATAC-seq references. **b** Benchmarking on excitatory neuron clusters. Left: heatmaps of chromatin marker regions of excitatory neurons detected from scATAC-seq references and the expressions of corresponding marker genes. Selected marker genes with highest fold changes are shown in the right panel. Right: the fold changes of enrichment between ATAC-seq defined marker regions and RNA-seq defined associated regions among excitatory neurons.

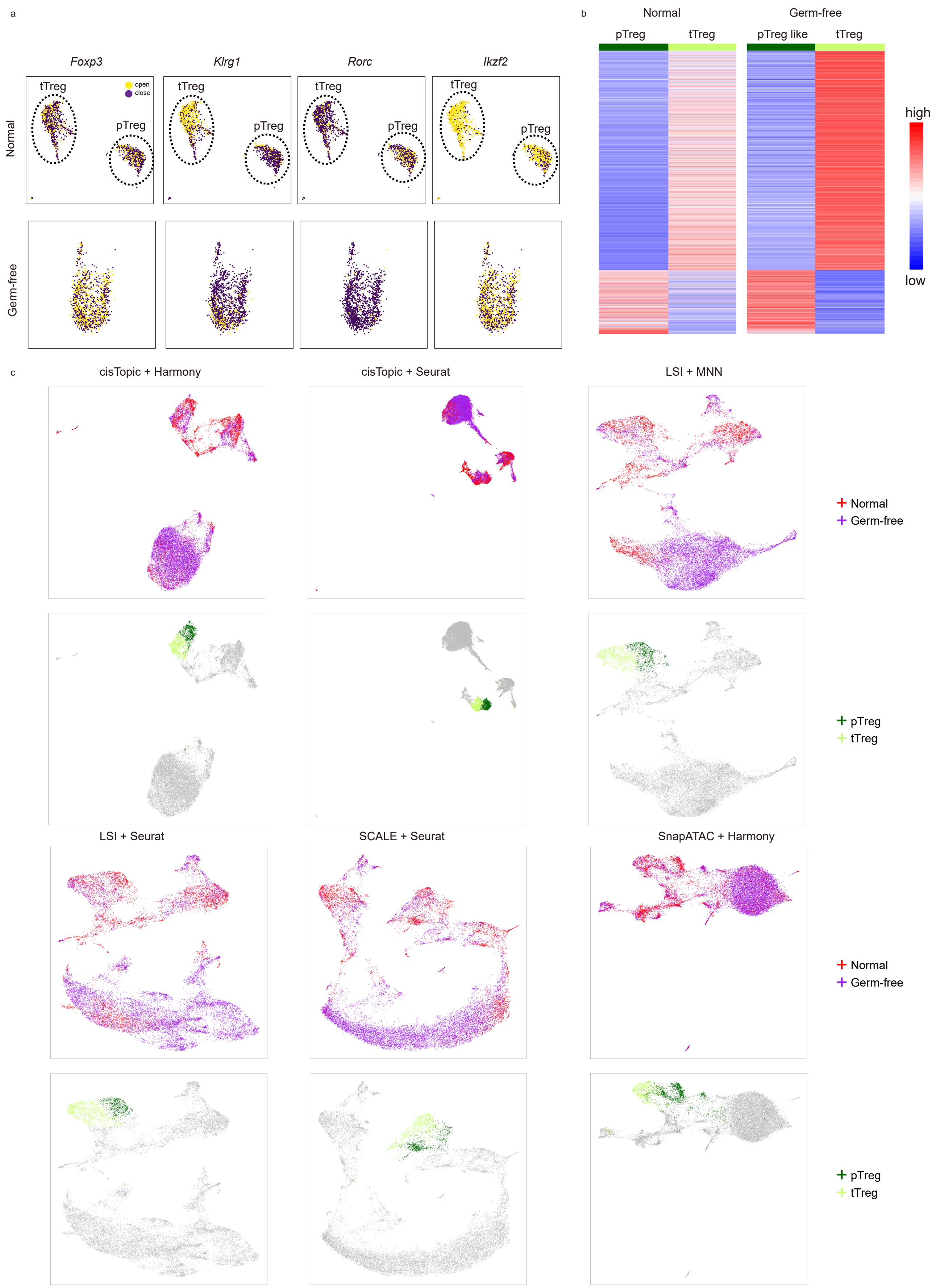


**Figure S8** supplementary figures for **Fig. 5**. **a** The accessibility of pTreg (*Foxp3*$^{open}$*Klrg1*$^{closed}$*Rorc*$^{open}$*Ikzf2*$^{closed}$) and tTreg (*Foxp3*$^{open}$*Klrg1*$^{open}$*Rorc*$^{close}$*Ikzf2*$^{open}$) marker gene promoters in normal and germ-free mouse. The pTreg and tTreg clusters can be inferred in normal mouse but not in germ-free mouse. **b** EpiConv finds two groups of Tregs in germ-free condition sharing common markers with pTreg and tTreg in normal condition.**c** Integrations of CD4+ T cells from normal and germ-free mouse by cisTopic + Hamony, cisTopic + Seurat, LSI + MNN, LSI + Seurat, SCALE + Seurat and SnapATAC + Harmony.

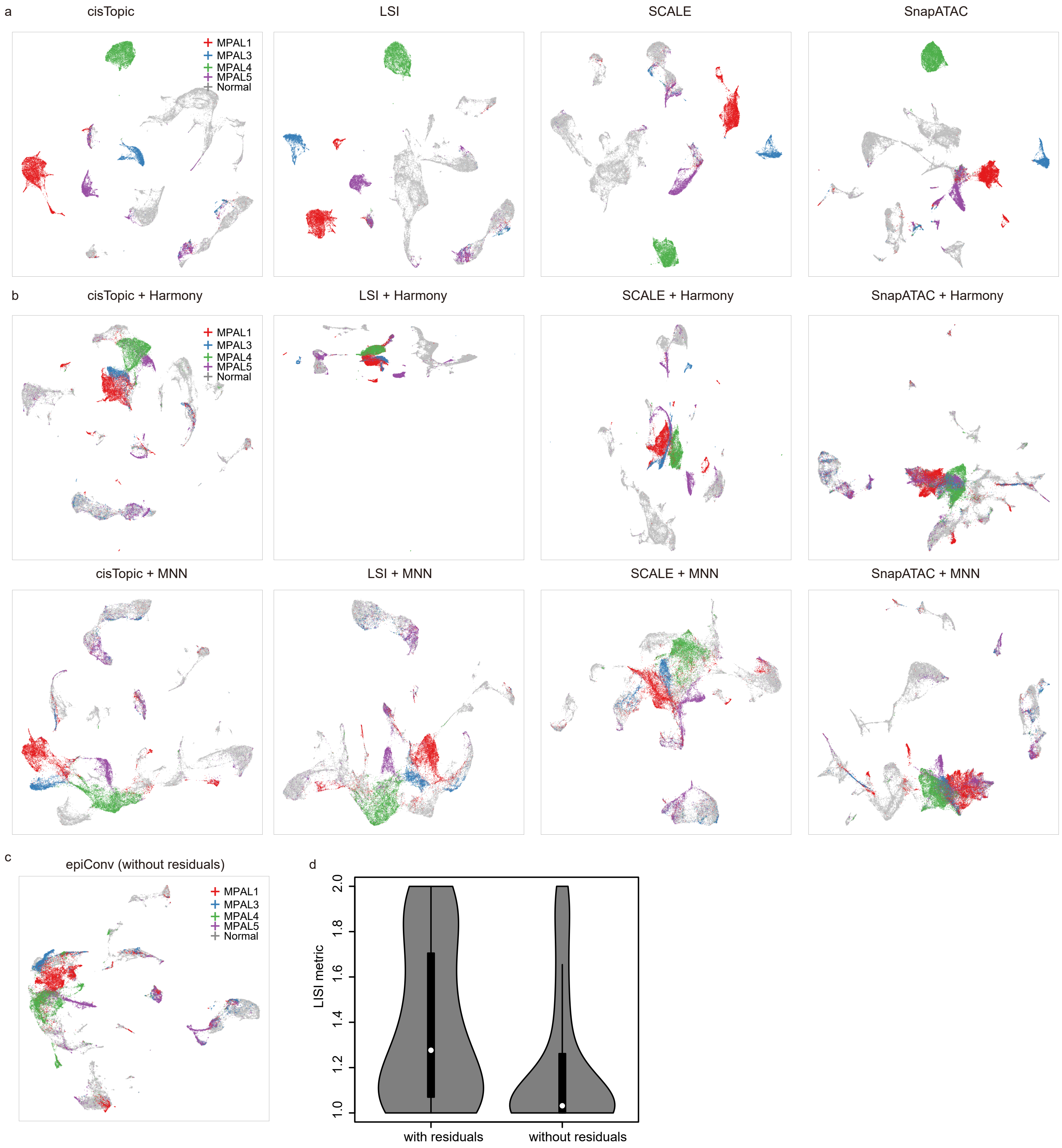


**Figure S9** Supplementary figures for **Fig. 6**. Low dimensional embedding of other methods. **a,b** CisTopic, LSI, SCALE and SnapATAC before (**a**) and after (**b**) batch correction by Harmony and MNN on Leukemia dataset.**c** Low dimensional embedding of epiConv without residuals. **d** Comparison of LISI metric between epiConv with and without residuals.

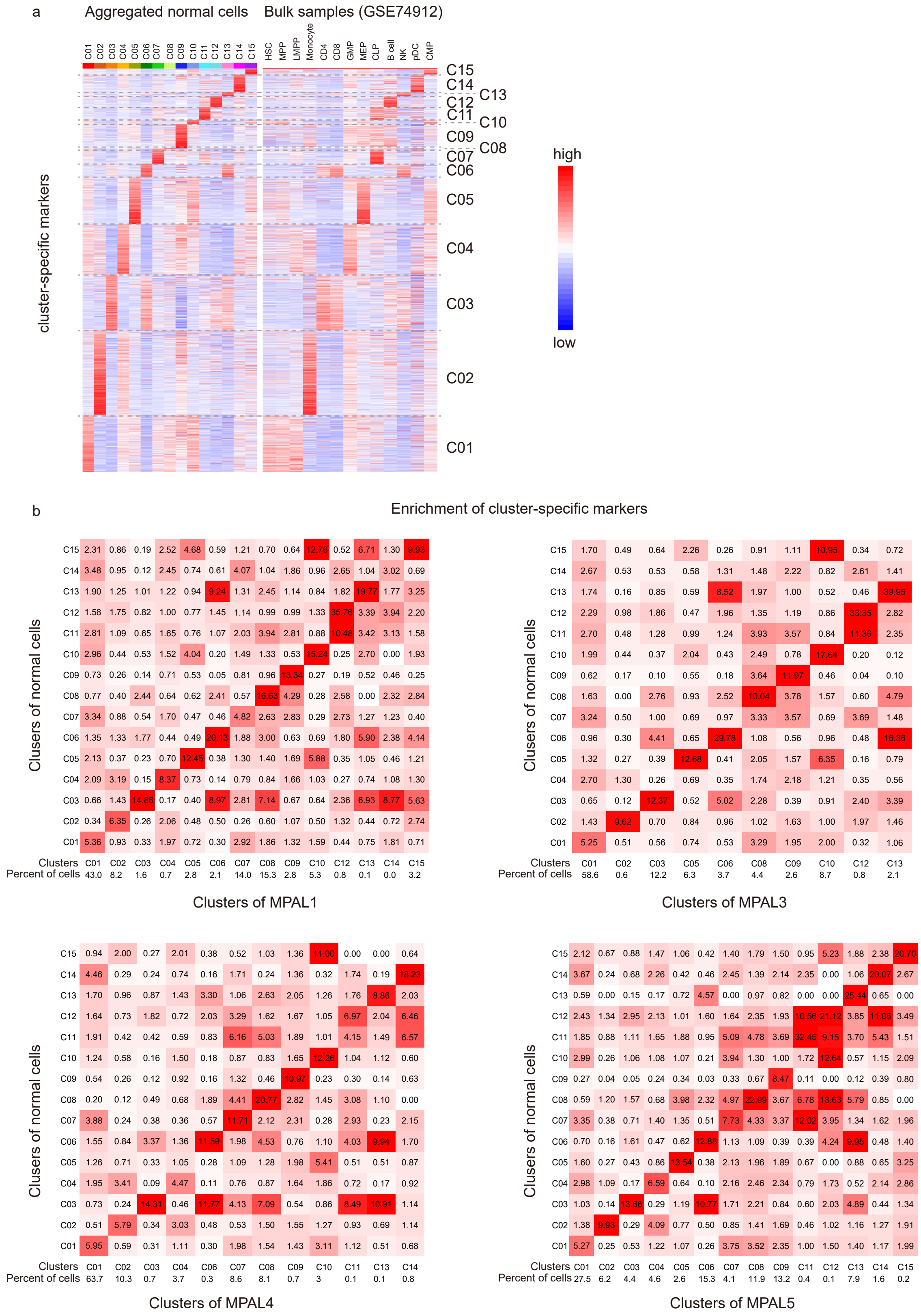


**Figure S10** Supplementary figures for **Fig. 6**. The identities of normal and malignant cells in Leukemia dataset. **a** Aggregated ATAC-seq profiles of normal cells (left) and bulk samples (right) on chromatin marker regions, showing biological identities of normal cells. **b** Fold change of enrichment between markers detected from normal cells and malignant cells, showing the similarities between normal and malignant clusters.

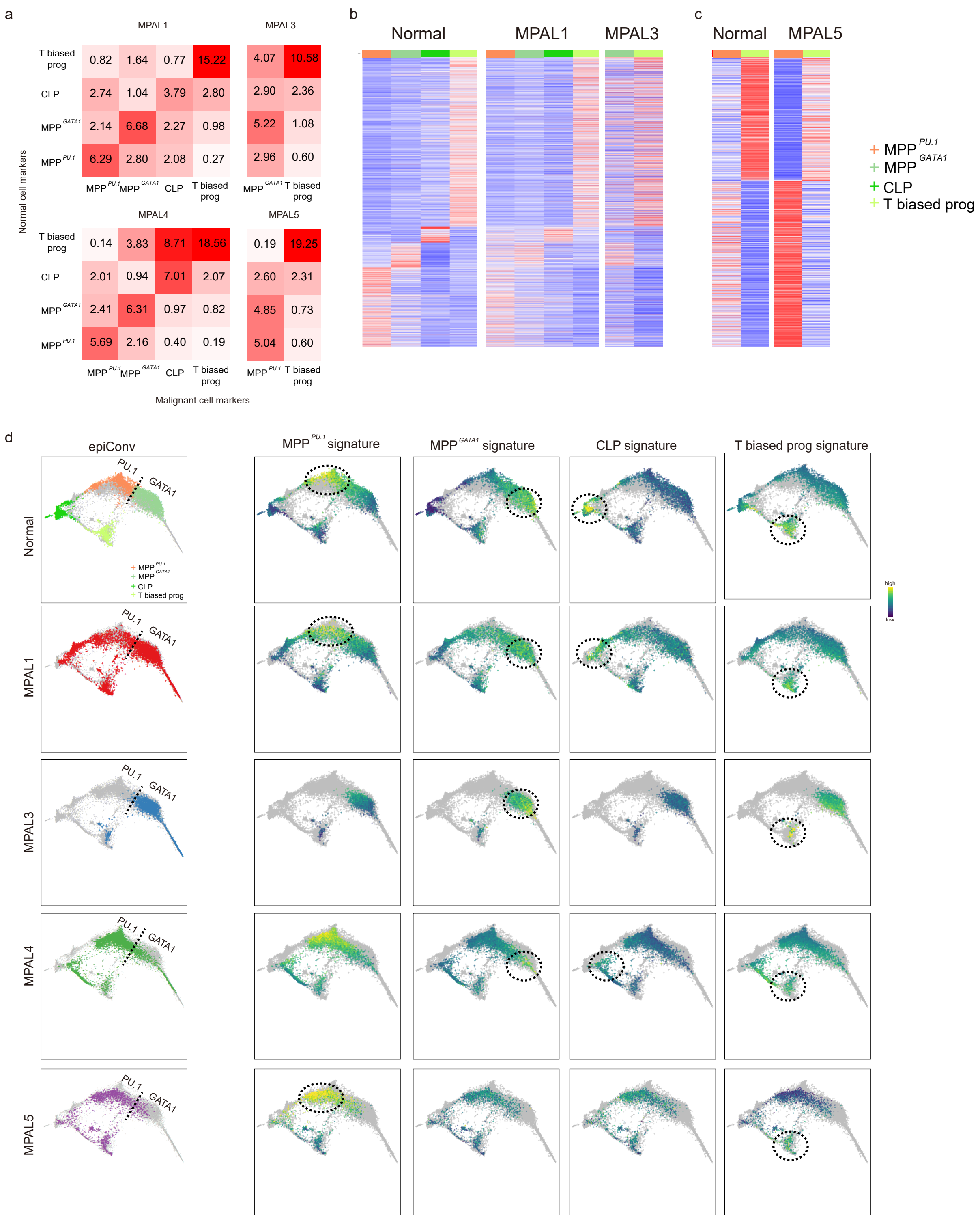


**Figure S11** Supplementary figures for **Fig. 6**. Conserved cluster-specific markers between normal and malignant samples. **a** Fold change of enrichment between markers detected from normal cells and malignant cells, showing the similarities between normal and malignant clusters. **b** Conserved markers between MPAL1, MPAL3 and normal cells. **c** Conserved markers between MPAL5 and normal cells. **d** Cluster-specific signatures of single cells from normal and malignant samples.

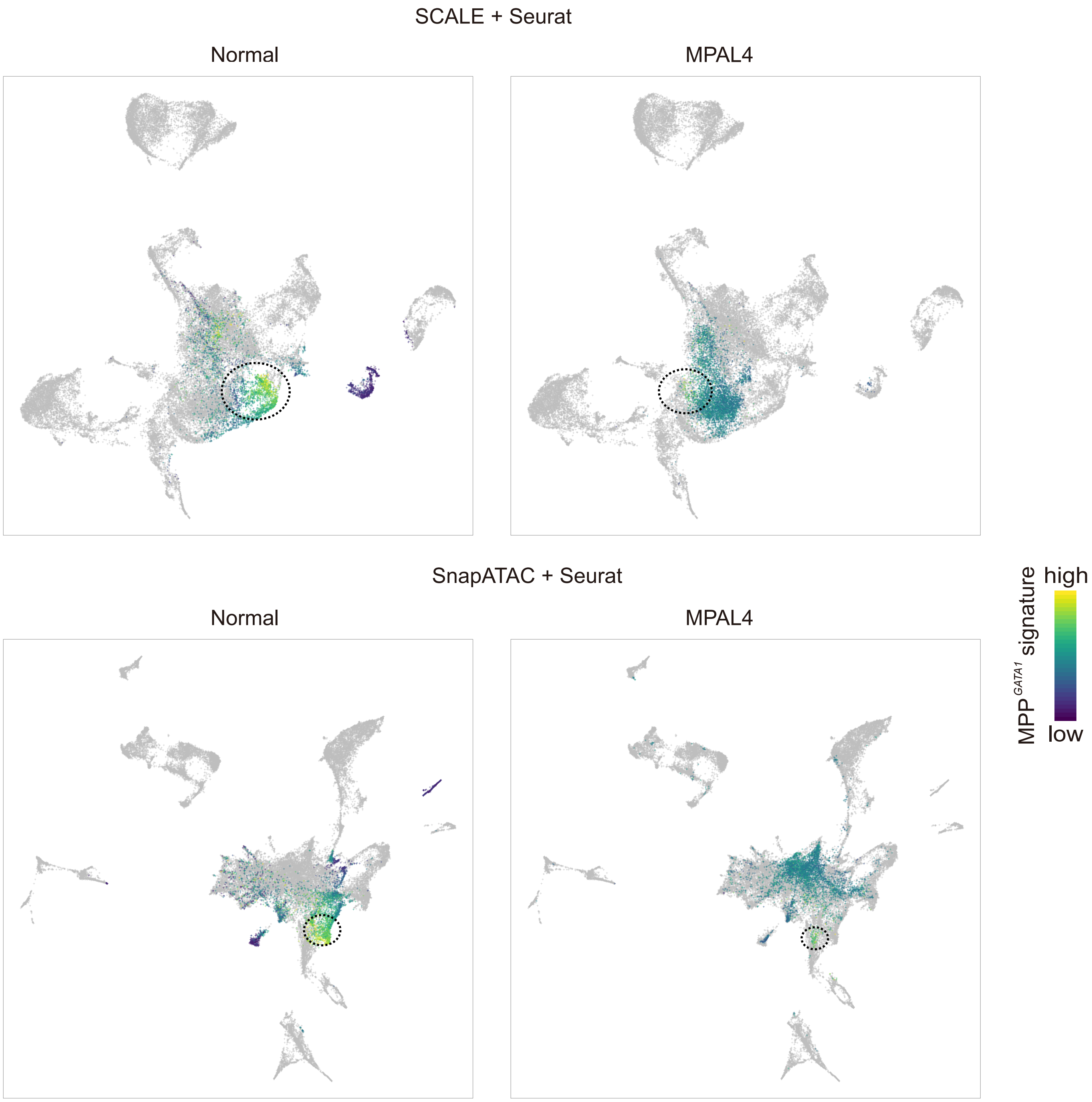


**Figure S12** supplementary figures for **Fig. 6**. Cluster-specific markers shared by normal cells and malignant cells of MPAL4 on the embeddings of SCALE + Seurat and SnapATAC+ Seurat. Dashed circles show cells with the highest signatures.

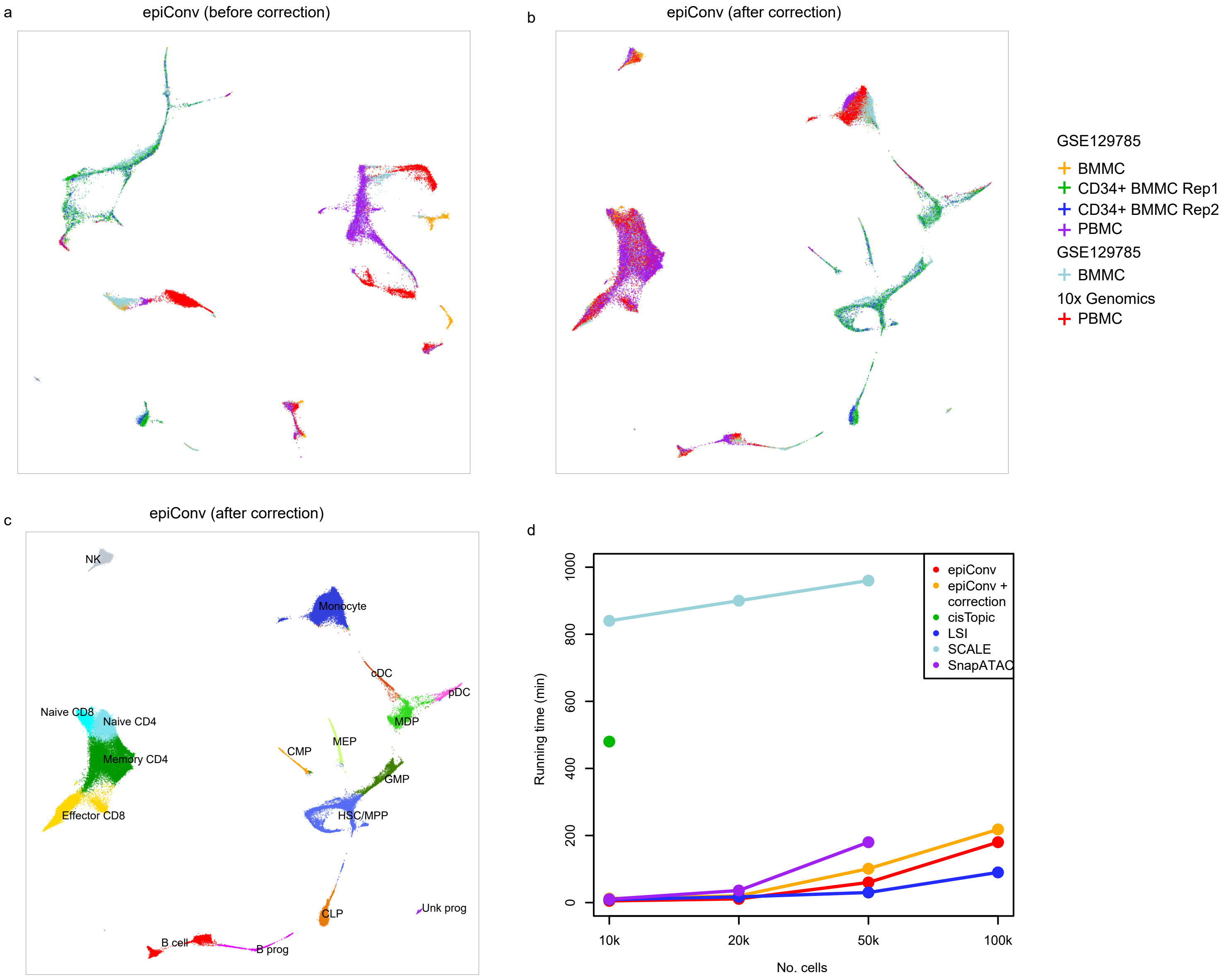


**Figure S13** Computational efficiency of epiConv on human bone marrow and blood mononuclear cells data (~100k cells). **a** Low dimensional embedding of epiConv before batch correction. **b,c** Low dimensional embedding of epiConv after batch correction colored by batch (**b**) and cell identities (**c**). HSC, hematopoietic stem cell; MPP, multipotent progenitor; GMP, granulocyte-macrophage progenitor; MDP, monocyte-DC progenitor; MEP, megakaryocyteerythroid progenitor; CLP, common lymphoid progenitor; CMP, common myeloid progenitor, Unk prog, unknown progenitor; cDC, conventional dendritic cell dendritic cell; pDC, plasmacytoid dendritic cell. **d** Running time of epiConv and other methods on dataset down-sampled to 10k, 20k, 50k and 100k cells. Running tests with more than 24 hours or exceeding memory limit are not shown.

# Supplementary Note

Here we describe a simple method to calculate the similarities between single cells of ATAC-seq data. First, assume that we have two distributions, $p_X$ and $p_Y$, where cells sample their insertion events from (the chromatin states of two types of cells). If we sample one insertion from $p_X$ and one insertion from $p_Y$, we have a new random variable that is 1 if two insertions occur in the same peak or 0 otherwise. Given random binary vectors *X* with *m* non-zero elements sampled from $p_X$ and *Y* with *n* non-zero elements sampled from $p_Y$, the dot product of *X* and *Y* can be considered as the sum of *m* x *n* variables and is determined by $p_X$ and $p_Y$ if *X* and *Y* are sparse. Thus, the $X \cdot Y/(m \cdot n)$ can be used to measure the similarity between *X* and *Y*. However, the value of $X \cdot Y$ is mainly affected by the peaks that have higher frequency. To balance the contribution of peaks with high frequency and low frequency, we weight each peak by $w = log_{10}(1 + inverse\ frequency\ of\ peak)$ following LSI, where peaks with higher frequency have lower weight. The observations from real data also support our assumption that the $X \cdot Y$ can be modeled as *m* x *n* random variables (**Fig. a** below).

In order to reduce the noise of similarity measurement, we adopt a bootstrap strategy. In *i*th bootstrap, we calculate $log_{10}\ (X_i \cdot Y_i) - log_{10}m - log_{10}n$, where we randomly sample some peaks from *X* and *Y* to generate $X_i$ and $Y_i$ (we sampled 20% peaks in each bootstrap and performed 15 bootstraps in this study), and use the mean of $log_{10}\ (X_i \cdot Y_i) - log_{10}m - log_{10}n$ across bootstraps as the similarity between cells.

Even after normalization by *m* x *n*, there are still some dependencies between the similarities and the library sizes. First, similarities between cells with small library sizes tends to have large variations (**Fig. b** below), which is consistent with our initial assumption as the mean value of fewer random variable has larger variation. Second, there are weak negative correlation between the similarity and library size (**Fig. b** below, coefficient ≈ -0.05 in most cases), as the *m* x *n* variables are not independent. We perform linear regression on log transformed similarities and $log_{10}m + log_{10}n$. The regression residuals are further divided by the corresponding standard deviations, calculated by data points with similar $log_{10}m + log_{10}n$. The regression residuals after variance stabilization are used as the similarity scores between two cells (**Fig. c** below).

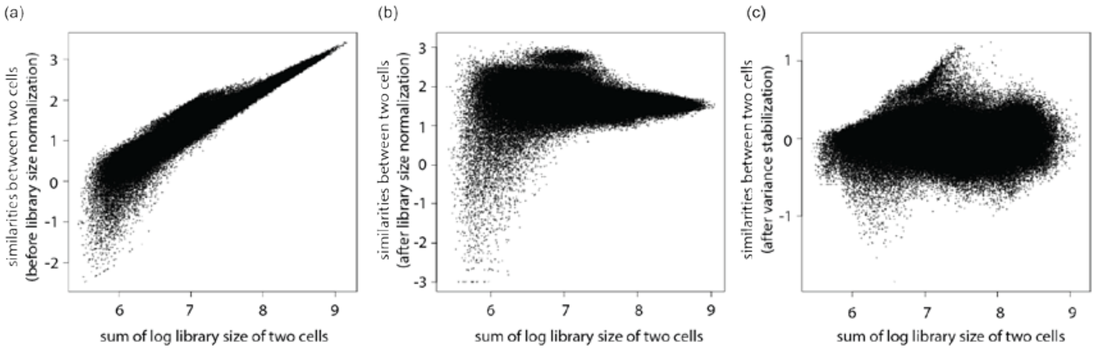

(a)
similarities between two cells (before library size normalization)
sum of log library size of two cells
(b)
similarities between two cells (after library size normalization)
sum of log library size of two cells
(c)
similarities between two cells (after variance stabilization)
sum of log library size of two cells
